# Palmitate induces integrated stress response and lipoapoptosis in trophoblasts

**DOI:** 10.1038/s41419-023-06415-6

**Published:** 2024-01-11

**Authors:** Prakash Kumar Sahoo, Chandan Krishnamoorthy, Jennifer R. Wood, Corrine Hanson, Ann Anderson-Berry, Justin L. Mott, Sathish Kumar Natarajan

**Affiliations:** 1 Department of Nutrition and Health Sciences, University of Nebraska-Lincoln, Lincoln, NE USA; 2 Department of Animal Sciences, University of Nebraska-Lincoln, Lincoln, NE USA; 3 College of Allied Health Professions Medical Nutrition Education, University of Nebraska Medical Center, Omaha, NE USA; 4 Department of Pediatrics, University of Nebraska Medical Center, Omaha, NE USA; 5 Department of Biochemistry and Molecular Biology, University of Nebraska Medical Center, Omaha, NE USA; 6 Department of Biochemistry, University of Nebraska-Lincoln, Lincoln, NE USA

**Keywords:** Fatty acids, Obesity

## Abstract

Maternal obesity increases the risk of childhood obesity and programs the offspring to develop metabolic syndrome later in their life. Palmitate is the predominant saturated free fatty acid (FFA) that is transported across the placenta to the fetus. We have recently shown that saturated FFA in the maternal circulation as a result of increased adipose tissue lipolysis in third trimester of pregnancy induces trophoblast lipoapoptosis. Here, we hypothesized that palmitate induces integrated stress response by activating mitogen-activated protein kinases (MAPKs), endoplasmic reticulum (ER) stress and granular stress and lipoapoptosis in trophoblasts. Choriocarcinoma-derived third-trimester placental trophoblast-like cells (JEG-3 and JAR) referred as trophoblasts were exposed to various concentrations of palmitate (PA). Apoptosis was assessed by nuclear morphological changes and caspase 3/7 activity. Immunoblot and immunofluorescence analysis was performed to measure the activation of MAPKs, ER stress and granular stress response pathways. Trophoblasts exposed to pathophysiological concentrations of PA showed a concentration-dependent increase in trophoblast lipoapoptosis. PA induces a caspase-dependent trophoblast lipoapoptosis. Further, PA induces MAPK activation (JNK and ERK) via phosphorylation, and activation of ER stress as evidenced by an increased phosphorylation eIF2α & IRE1α. PA also induces the activation of stress granules formation. Two pro-apoptotic transcriptional mediators of PA-induced trophoblast lipoapoptosis, CHOP and FoxO3 have increased nuclear translocation. Mechanistically, PA-induced JNK is critical for trophoblast lipoapoptosis. However, PA-induced activation of ERK and stress granule formation were shown to be cell survival signals to combat subcellular stress due to PA exposure. In conclusion, PA induces the activation of integrated stress responses, among which small molecule inhibition of JNK demonstrated that activation of JNK is critical for PA-induced trophoblast lipoapoptosis and small molecule activation of stress granule formation significantly prevents PA-induced trophoblast lipoapoptosis.

## Introduction

The obesity epidemic in the United States and worldwide extends its potential health hazard to pregnant people. Obesity, and the associated metabolic dysfunction has become a major public health concern among women of reproductive age [1]. Maternal obesity is also positively associated with increased risk of adverse pregnancy outcomes to the mother and offspring including gestational diabetes, pre-eclampsia, maternal inflammation, intrauterine growth retardation and large-for-gestational-age infants [2, 3]. There is accumulating evidence that maternal obesity impacts the metabolic health of newborns and increases the lifetime risk of offspring developing metabolic syndrome including obesity, diabetes and cardiovascular diseases [4–6].

Placenta, ‘the third brain’ which develops along with the fetus, plays a crucial role in fetal development and health. Trophoblasts, an important cell type in the placenta, are essential for nutrient supply for the developing fetus. Several reports have suggested that maternal obesity alters the placental architecture and negatively impacts fetal growth and development [7–10]. Placental lipid accumulation is also dramatically increased in obese mothers as compared to lean mothers [11]. A recent study obtained obese placenta from the first trimester of pregnancy and showed increased lipid accumulation and altered expression of major lipid metabolic genes in the placenta [9]. The expression of mitochondrial fatty acid oxidation genes and fatty acid esterification were downregulated; and increased expression of enzymes involved in the peroxisomal fatty acid oxidation in the placenta to the fetal compartment, and increased peroxisomal fatty acid oxidation is known to induce oxidative stress and subcellular stress in the liver [9, 12].

Palmitate, a predominant circulating lipotoxic saturated free fatty acid (FFA) is elevated in obese women [13, 14]. Maternal obesity is also associated with increased circulating triglycerides, cholesterol and insulin [9]. We have recently demonstrated that pathophysiological concentrations of saturated free fatty acids palmitate and stearate-induced trophoblast lipoapoptosis [14]. However, the underlying mechanism of trophoblast apoptosis remains unidentified. We hypothesize that palmitate induces an integrated stress response by activating mitogen-activated protein kinases (MAPKs), endoplasmic reticulum (ER) stress and granular stress and trophoblast lipoapoptosis. In the present study, we measured the activation of cellular stresses that promote cell survival.

## Materials and methods

### Materials

All chemicals and buffers were of analytical grade and purchased from ThermoFisher Scientific (Massachusetts, USA). Palmitic acid (A3803) and fatty acid free bovine serum albumin (BSA, P5585) were obtained from MilliporeSigma, St. Louis, MS. 15-deoxy-Δ12,14-Prostaglandin J2 (PGJ2) and GSK2606414 (GSK260) were purchased from Cayman, Ann Arbor, MI. SP600125 (JNKi), STF-083010 (IRE1αi, endonuclease inhibitor), Salubrinal (eIF2αi, dephosphorylation inhibitor), U0126 (ERKi, MEK1/2 or ERK1/2 Inhibitor) were obtained from Selleck Chemicals, Houston, TX.

### Antibodies

Primary antibodies against p-eIF2α (# 3388), eIF2α (# 5324), p-JNK (# 9251), JNK (# 9252), IRE1α (# 3294), p-ERK1/2 (# 9109), ERK1/2 (# 4695), CHOP (# 2895), Bip (# 3183), Bim (# 2933), PARP (# 9542), HDAC1(# 34589), and Calnexin (# 2679) were purchased from Cell Signaling Technologies, MA, USA. Phospho-IRE1α antibody (# ab48187) and ATF-6 (#ab122897) was obtained from abcam (Cambridge, UK) and Actin antibody (# A-5441) was from MilliporeSigma, MA. PUMA (sc-28226) was obtained from Santa Cruz Biotechnology (SCBT, TX, USA). Calreticulin (NBP1-47158) was purchased from Novus biologicals (CO, USA). Peroxidase-conjugated secondary antibodies were obtained from Jackson ImmunoResearch lab (PA, USA).

### Cell culture

HTR-8/SVneo (HTR-8) (CRL-3271), normal human immortalized first-trimester placental trophoblast-like cells, and choriocarcinoma-derived third-trimester placental trophoblast-like cells (JEG-3 and JAR: referred as trophoblasts) (HTB-36 & HTB-144 respectively) were used and obtained from ATCC. HTR-8 and JAR were cultured in DMEM (Corning) supplemented with 10% fetal bovine serum (Gibco) and antibiotics. JEG-3 cells were maintained in MEM (Corning) supplemented with 10% fetal bovine serum (Gibco) and antibiotics. Cells were maintained at 37^0 ^C in a 5% CO2 humidified incubator and passaged regularly (usually 3-4 days). These trophoblast-like cells were referred as trophoblasts in rest of the manuscript.

### Fatty acid preparation and cell treatment

Fatty acid (palmitate, PA, MilliporeSigma) was prepared by dissolving palmitate in Isopropanol to a stock concentration of 80 mM. For cell treatment, the fatty acid stock was diluted to appropriate concentrations in 1% BSA containing complete growth medium and incubated at 37 °C for 30 min for fatty acid conjugation to BSA. 1% BSA containing media was prepared by dissolving BSA in complete growth medium at room temperature and further incubating at 37 °C for 20 min as described [13]. Pathophysiological and physiologically achievable concentration range of 200–800 μM palmitate were used for the present study and vehicle (Veh) treatment were <1% isopropanol in 1% BSA containing medium.

### Characterization of apoptosis

Apoptosis was analyzed via assessing percent apoptotic nuclei and caspase 3/7 activity, which represent structural and biochemical markers of apoptosis, respectively. Percent apoptotic nuclei was quantified by characteristic nuclear morphology and visualized with the treatment of DNA binding fluorescent dye, DAPI (4’, 6-diamidine-2-phenylindole dihydrochloride) [14]. Briefly, cells were stained with DAPI (5 µg/ml) for 10–15 min at 37 °C. Apoptotic cells, characterized by condensed and fragmented nuclei were counted and presented as percent of total nuclei. Experiments were performed in triplicates and at least 200 cells were counted per well. Caspase 3 and 7 activity was analyzed using rhodamine 110 bis-(N-CBZ-l-aspartyl-l-glutamyl-l-valyl-aspartic acid amide) (Z-DEVD-R110) caspase substrate according to manufacturer’s instructions (Promega, Madison, WI #G7791). Briefly, cells with active caspase 3/7 enzyme cleaves the substrate Z-DEVD releasing rhodamine which was quantified spectrofluorometrically using Biotek Synergy plate reader [14]. The experiment was performed in quadruplicate and were reported as fold change compared to vehicle-treated cells.

### Flow cytometry

JEG-3 cell viability was measured using simultaneous measurement of propidium iodide (PI; 1 µg/ml; BD Biosciences 556463) uptake and Annexin V-FITC (BD Biosciences 556419) binding of cell membrane. Cells positive for Annexin V only or both Annexin V and PI were considered as early and late apoptotic respectively. Flow cytometry was performed using Beckman Coulter CytoFLEX and data was analyzed using CytExpert software.

### Viability analysis with intracellular ATP measurement

Commercially available CellTiter-Glo 2.0 Cell Viability kit (Promega, G9242) was used to measure intracellular ATP (according to manufacturer instructions). Briefly, cells were plated in a 96-well plate at 8000 cells/well and treated accordingly. After 24 h, CellTiter-Glo reagent was added in an equal volume to promote cell lysis. ATP was quantified via luminescence measurement using Biotek Synergy plate reader.

### Cell lysate preparation and Immunoblot analysis

Cells were washed with ice cold phosphate-buffered saline (PBS, 1X) once and scraped from the plate using cell lysis buffer (50 mM Tris pH 7.4, 150 mM NaCl, 1 mM EDTA, 1 mM DTT, 1 mM Na_3_Vo_4_, 1 mM PMSF, 100 mM NaF, and 1% Triton x-100) supplemented with Halt protease and phosphatase inhibitor cocktail (# 78440, Thermo Fisher, MA. USA). Collected cells were incubated on ice for 30 min to facilitate cell lysis, centrifuged at 14,000X RPM for 20 min at 4 °C and the clarified supernatant containing protein was collected. Total protein quantification was performed using modified Lowry method using Pierce 660 nm protein assay reagent (# 22660, Thermo Fisher, MA, USA). A total of 20–30 μg protein were resolved on 10% or 12% polyacrylamide gel containing SDS and further transferred onto nitrocellulose membrane (Bio-Rad, CA, USA) using a Bio-Rad wet transfer system. Non-specific protein blocking was performed using either 5% skim milk or BSA in Tris-buffered saline containing 0.1% Tween 20 (TBS-T) and incubated with primary antibody (1:1000) solution at 4 °C overnight. The membranes were washed three times with TBS-T and incubated with HRP-conjugated secondary antibody (1:5000 dilution) solution for 2 h at room temperature. Protein bands were visualized using chemiluminescent ECL substrate (# 170-5061, Bio-Rad; # NEL104001, PerkinElmer, MA, USA; or # A38554, Thermos Scientific, MA, USA) using Bio-Rad Chemidoc imaging system.

### Isolation of nuclear proteins

Cells were washed with PBS and scraped using Buffer A containing 10 mM HEPES, 10 mM KCl, 0.1 mM EDTA, 0.1 mM DTT, 0.5% nonidet-P40 substitute (MilliporeSigma) with protease inhibitor (Roche) and incubated on ice for 10 min. Cell lysate was centrifuged at 15,000 × *g* for 3 min and supernatant was separated (which contains cytosolic protein). To the pelleted nuclear content, Buffer B containing 20 mM HEPES, 0.4 M NaCl, 1 mM EDTA, 0.05 mM DTT, and 10% glycerol with protease inhibitor was added and incubated on ice with intermittent vortexing for 40 min. The nuclear content was centrifuged at 15,000 × *g* for 5 min and supernatant containing nuclear protein was collected [13, 15] and stored at −80 °C until used.

### Immunofluorescence analysis

Cells were grown on collagen coated coverslips and treated appropriately. At the end of experiment, cells were washed twice with PBS and fixed using 3% paraformaldehyde (Electron Microscopy Sciences, PA, USA) in PBS with 100 mM PIPES, 3 mM MgSO_4_ and 1 mM EGTA for 20 min at 37 °C. Then cells were washed 3 times with PBS and mild permeabilization was performed for 5 min at room temperature using 0.3% Tween 20 in PBS. After permeabilization, cells were washed with PBS three times and blocked for 60 min at 37^0^C using PBS with 5% glycerol, 5% goat serum and 0.01% sodium azide. Primary antibody incubation was performed using the above buffer (1:100 or 1:250 dilution) at 4 °C overnight. Next, cells were washed three time with PBS and incubated with Alexa conjugated secondary antibody (Invitrogen, MA USA) for 60 mins at 37 °C. Cells were washed with PBS once and deionized H_2_O once and counterstained with nuclear stain DAPI, further washed with PBS twice and mounted on microscope slides using fluoromount-G (Electron Microscopy Sciences, PA, USA). Images were acquired using Nikon A1R-Ti2 confocal system and optimized using ImageJ software (National Institute of Health).

### Quantitative real-time polymerase chain reaction (qRT-PCR) analysis

Cells were lysed in the well using TRIzol reagent (# 15596018, Invitrogen, MA, USA) and RNA was isolated according to manufacturer’s instruction. Isolated total RNA was quantified and checked for purity using Biotek Synergy plate reader. One microgram of total RNA was reverse transcribed into cDNA using random hexamers, RNase OUT (Invitrogen, MA, USA), dNTPs and Murine-MuLV reverse transcriptase (NEB, MA, USA). qRT-PCR was performed using Light Cycler 480 SYBR Green I Master mix (# 04707516001, Roche, Basel, Switzerland) according to manufacturer instructions in a Bio-Rad CFX Connect real-time system. Target genes and primer list provided in Table 1.

**Table 1:** List of primers used.

Target gene	Forward primer	Reverse primer	Product length
CHOP	ATGGCAGCTGAGTCATTGCCTTTC	AGAGACAGGGTCAAGAGTGGTGAA	265 bp
XBP1	AAACAGAGTAGCAGCTCAGACTGC	TCCTTCTGGGTAGACCTCTGGGAG	3523 bp
18S rRNA	CGTTCTTAGTTGGTGGAGCG	CGCTGAGCCAGTCAGTGTAG	212 bp
GAPDH	AATCCCATCACCATCTTCCA	TTCACACCCATGACGAACAT	413 bp

### XBP1 mRNA splicing assay

One micrograms of RNA was converted into cDNA and further diluted to 1:3 (JEG-3 and JAR) or 1:10 (HTR-8) [16]. The diluted samples were further PCR amplified using *XBP1* specific primers (Table 1). Next, 8 µl of PCR amplified XBP1 was restriction digested with 20 U of *PstI* (catalog no, R0140, NEB, MA, USA) in 1 µl of NEB buffer at 37 °C for 2 h. The restriction digested products were separated on 1% Agarose gel stained with ethidium bromide. The unspliced XBP1 of 474 bp upon digestion produces fragments of 296 bp and 183 bp, while spliced XBP1 lacking restriction enzyme site is of 448 bp length. GAPDH was used as control.

### Statistical analysis

The data were analyzed using two-way analysis of variance (ANOVA) with Bonferroni post-hoc test for comparison between multiple groups and Student’s *t*-test for comparison between two groups using GraphPad Prism 9. The data were plotted as means and standard errors of means (SEM) using GraphPad Prism.

## Results

### Palmitate induces trophoblast lipoapoptosis

**Fig. 1:**
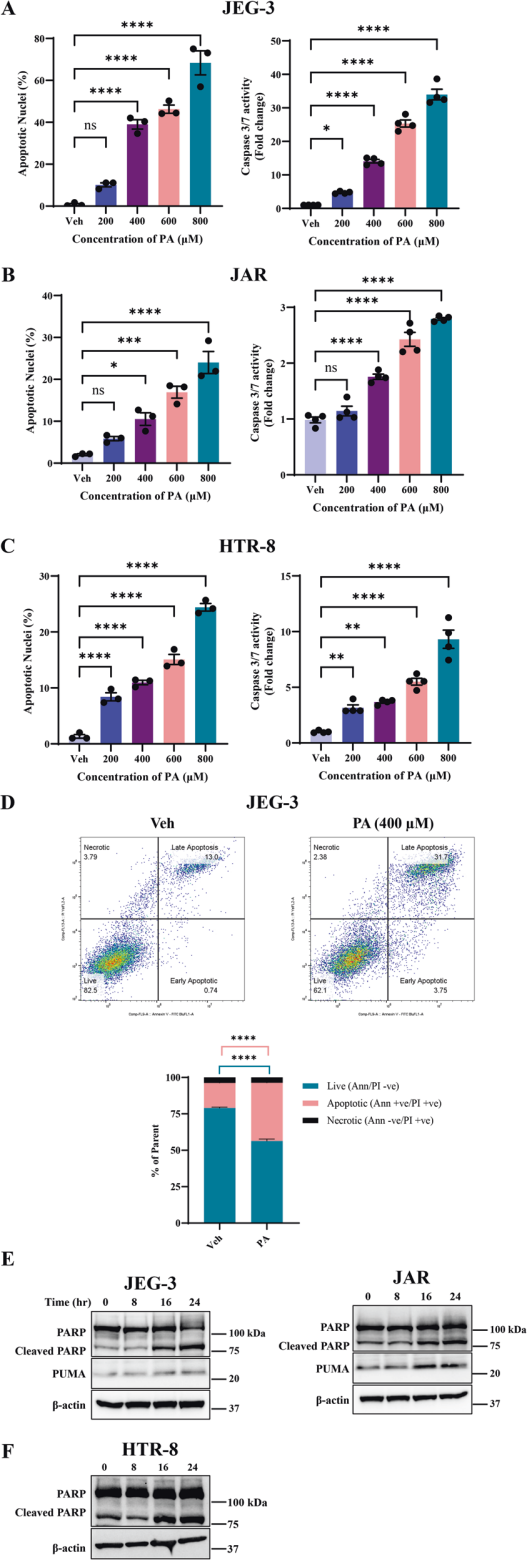
Palmitate (PA)-induced trophoblast lipoapoptosis. **A** JEG-3 were treated with 200–800 μM of PA for a period of 24 h and apoptosis were analyzed. **B** JAR cells were treated with 200–800 μM of PA for a period of 24 h and apoptosis were analyzed. **C** HTR-8 cells were treated with 200–800 μM of PA for a period of 24 h and apoptosis were analyzed. **D** Flow cytometric analysis of JEG-3 cells treated with PA (400 µM) for 24 h. PA treatment showed increased late apoptotic cells and decreased number of live cells. **E** JEG-3 and JAR proteins were collected at different times for western blot analysis. Increased levels of cleaved PARP and increased PUMA were observed as an indicator of apoptosis induction with increasing exposure time of PA. **F** HTR-8 cells also showed increased levels of cleaved PARP after 16–24 h of PA exposure. Data presented as mean ± SEM, *n* = 4. *p < 0.05, **p < 0.01, ***p < 0.001, ****p < 0.0001 compared to vehicle-treated cells.

Palmitate (PA) is the most common saturated free fatty acid observed to be increased in the circulation during obesity and we used PA as the mediator of trophoblast lipoapoptosis. We treated trophoblasts, JEG-3 and JAR cells with increasing concentrations of PA (200–800 μM) and assessed lipoapoptosis after 24 h by nuclear morphological changes using DAPI staining and caspase 3/7 activation. Both JEG-3 and JAR cells showed an increase in percent apoptotic nuclei (measurement of DNA condensation and fragmentation) starting from the lower concentration of PA (400 μM) when compared to vehicle (Veh) treated cells (Fig. 1A, B). Higher concentrations of PA (600–800 μM) also showed significant increase in percent apoptotic nuclei compared to vehicle and 400 μM of PA-treated trophoblasts (Fig. 1A, B). However lowest concentration of PA (200 μM) showed only a trend towards increase in percent apoptotic nuclei (Fig. 1A, B). We simultaneously assessed caspase 3/7 activity with PA treatment and observed a significant increase in caspase 3/7 activity with increasing concentration of PA (200–800 μM) in trophoblasts. We next used HTR-8, an immortalized trophoblasts from first-trimester placenta and treated the cells with increasing concentrations of palmitate (200–800 µM). Similar to JEG-3 and JAR cells, treatment of PA to HTR-8 cells significantly increased both percent apoptotic nuclei and caspase 3/7 activity compared to vehicle-treated cells (Fig. 1C). To further delineate whether PA induces any non-apoptotic cell death, JEG-3 cells were treated with PA (400 µM) for 24 h and cell death was analyzed using flow cytometry. We used propidium iodide (PI) as an indicator of cell membrane integrity loss and FITC-Annexin V which binds to phosphatidylserine residues present only on apoptotic cells to measure PA-induced lipoapoptosis. We observed a simultaneous decrease in percent live cells and increase in percent apoptotic cells with PA treatment compared to vehicle treatment (Fig. 1D). Cells undergoing secondary necrosis remained similar for both vehicle and PA-treated groups (Fig. 1D). Next, we used immunoblot analysis of the downstream mediator and pro-apoptotic mediators of caspase activation during trophoblast lipoapoptosis by measuring the levels of poly (ADP-ribose) polymerase (PARP) and p53 upregulated modulator of apoptosis (PUMA), respectively. Activated caspase can cleave PARP into cleaved PARP, which is a hallmark of lipoapoptosis. We observed evidently increased levels of both cleaved PARP and PUMA at 16 and 24 h post treatment with palmitate in both JEG-3 and JAR cells (Fig. 1E). Similarly, in HTR-8 cells, immunoblot analysis of PARP levels with the treatment of PA showed marked increase in the levels of cleaved PARP after 16 and 24 h compared to vehicle-treated cells (Fig. 1F). These data support that, saturated free fatty acid, palmitate induces trophoblast lipoapoptosis, in vitro.

### Palmitate induces a caspase-dependent trophoblast lipoapoptosis

**Fig. 2:**
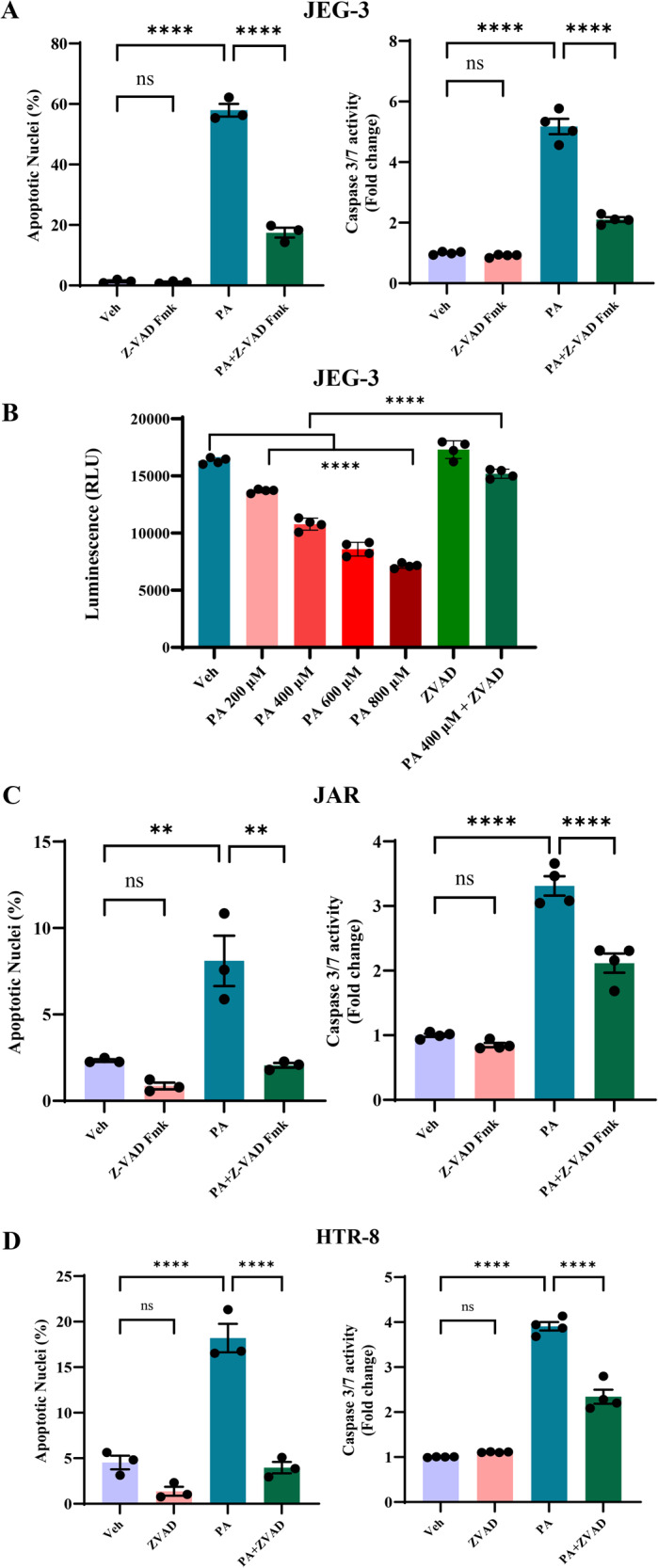
Palmitate induces a caspase-dependent trophoblast lipoapoptosis. **A** JEG-3, **C** JAR cells and **D** HTR-8 were treated with either vehicle (Veh), palmitate (PA), Z-VAD-fmk, or PA + Z-VAD for 24 h. Percent apoptotic nuclei (left panel) and caspase 3/7 activity (right panel) were significantly elevated with PA-treated cells. Cotreatment of PA and Z-VAD-fmk prevents PA-induced trophoblast lipoapoptosis. **B**. JEG-3 cells were treated with either Veh, PA (200–800 µM), Z-VAD-fmk or PA (400 µM) + Z-VAD-fmk for 24 h and viability was analyzed using CellTiter-Glo 2.0. PA treatment significantly reduced intracellular ATP levels, while co-treatment of Z-VAD-fmk with PA (400 µM) restored intracellular ATP levels, compared with vehicle-treated cells (Veh). Data presented as mean ± SEM, *n* = 4. *P < 0.05, **p < 0.01, ***p < 0.001, ****p < 0.0001 compared to vehicle-treated cells or PA-treated cells.

We tested the critical role of caspase activation in trophoblast lipoapoptosis using pan-caspase inhibitor, Z-VAD-fmk. We treated trophoblasts with 400 µM of palmitate and 50 µM of Z-VAD-fmk for 24 h and analyzed lipoapoptosis. Similar to the data in Fig. 1, PA-induced significant increase in percent apoptotic nuclei levels and caspase 3/7 activation in JEG-3, JAR and HTR-8 cells suggesting trophoblast lipoapoptosis (Fig. 2A, C, D). Cotreatment of PA and Z-VAD-fmk significantly protected against PA-induced trophoblast lipoapoptosis (Fig. 2A, C, D). Treatment of Z-VAD-fmk alone to JEG-3, JAR or HTR-8 cells did not induce apoptosis and are similar to vehicle-treated cells. We also performed cell viability using intracellular ATP quantification to measure metabolically healthy cells. JEG-3 cells treated with PA (200–800 µM) showed a significant decrease in intracellular ATP levels compared to vehicle-treated cells (Fig. 2B). Further, cotreatment of PA, 400 µM and Z-VAD-fmk showed significantly restored intracellular ATP levels. Z-VAD-fmk alone did not change intracellular ATP levels and the levels were comparable to vehicle-treated cells (Fig. 2B). Further, JEG-3 cells showing increased DAPI staining with increasing concentration of PA and cotreatment of PA (400 µM) and Z-VAD-fmk dramatically decreased DAPI positive cells (Supplementary Fig. S1). Together, palmitate, a saturated FFA induces a caspase-dependent trophoblast lipoapoptosis.

### Palmitate induces endoplasmic reticulum (ER) stress in trophoblasts

**Fig. 3:**
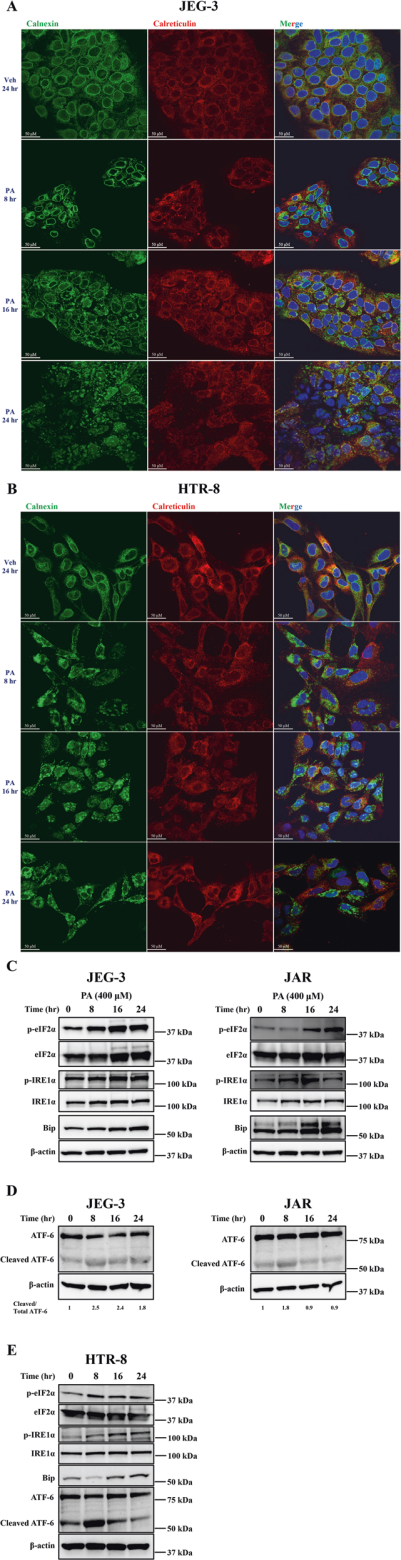
Palmitate induces endoplasmic reticulum (ER) stress in trophoblasts. **A** JEG-3, **B** HTR-8 cells treated with palmitate (PA) at 400 µM for 8–24 h showed altered ER morphology as seen with calnexin and calreticulin staining compared to vehicle-treated trophoblasts. **C**, **D** Immunoblot analysis showed an increase in the levels of phosphorylated IRE1α, eIF2α and active form of ATF-6 (cleaved ATF-6) after 16–24 h of PA-treated JEG-3 and JAR cells compared to vehicle-treated cells. **E** Immunoblot analysis of HTR-8 cells showed activation of all three arms of ER stress pathway (phosphorylation of IRE1α, eIF2α and cleaved ATF-6) and increased expression of ER chaperone, BIP in PA-treated cells compared to vehicle. Beta-actin was used as loading control and remained unchanged. The images are representative images. Scale bar represents 50 µM.

Calnexin, an ER resident glycoprotein is known to help in protein folding and we employed immunofluorescent analysis of calnexin in trophoblasts after 16–24 h of palmitate treatment. Calnexin showed perinuclear staining pattern in vehicle-treated cells (Fig. 3A, B). In contrast, palmitate-treated trophoblast showed distorted expression pattern suggesting ER morphological changes during palmitate-induced trophoblast lipoapoptosis (Fig. 3A, B). However, we did not observe any changes in the levels of calnexin protein when measured by immunoblot analysis (Supplementary Fig. S3A). These data suggest that ER morphological changes were observed in trophoblasts with the treatment of palmitate and led to our hypothesis that the mechanism of palmitate-induced trophoblast lipoapoptosis involves the activation of ER stress.

ER stress results with the activation of three arms of unfolded protein response (UPR) like inositol requiring enzyme 1 alpha (IRE1α), protein kinase R-like ER kinase (PERK) and activating transcription factor 6 (ATF-6). We first measured levels of phosphorylated forms of eukaryotic initiation actor 2 alpha (eIF2α) as a downstream target of activated PERK using immunoblot analysis. Treatment of PA at 400 µM to JEG-3 or JAR cells resulted in the activation of eIF2α via phosphorylation after 8–24 h (Fig. 3C). Similarly, the levels of phosphorylated IRE1α were also increased after 16 h of PA treatment (400 µM). The levels of total eIF2α and total IRE1α were unchanged in PA and vehicle-treated cells. Further, ER stress is known to upregulate the expression of ER chaperones like Bip/GRP78. We next examined the levels of Bip/GRP78 with the treatment of PA at 400 µM (0–24 h) and observed that the expression of Bip was upregulated after 16–24 h in both trophoblasts (JEG-3 and JAR) tested compared to vehicle-treated cells (Fig. 3C). In response to ER stress, the third arm of UPR governed by ATF-6 is trafficked to Golgi, cleaved by proteases, and then translocate to nucleus to act as transcription factor for UPR associated proteins. Immunoblot analysis revealed presence of cleaved ATF-6 in JEG-3 cells and showed decreased levels of full-length ATF-6 at 8 and 16 h and went back to control (Veh) level by 24 h. The levels of cleaved ATF-6 levels were increased after 8–24 h of PA treatment compared to vehicle-treated cells as indicated by the ratio of cleaved ATF-6 and total ATF-6 (Fig. 3D). In JAR cells, we observed a subtle increase in cleaved ATF-6 with the treatment of PA after 8 h compared to control (Fig. 3D). We also measured the activation of ER stress in HTR-8 cells with the treatment of PA and showed a similar activation of eIF2α and IRE1α via phosphorylation after 8–24 h (Fig. 3E). HTR-8 cells also showed a dramatic increase in the levels of cleaved ATF-6 after 8 h of PA treatment and Bip expression was upregulated after 16–24 h of PA treatment compared to vehicle-treated cells (Fig. 3E). Actin levels were unchanged among different treatment conditions suggesting the equal loading of protein. Our data suggest that treatment of PA to trophoblasts showed activation of IRE1α and PERK arms of ER stress.

### PA activates XBP splicing and nuclear translocation of C/EBP homologous protein (CHOP) and forkhead family of transcription factor class O3 (FoxO3) in Trophoblasts

**Fig. 4:**
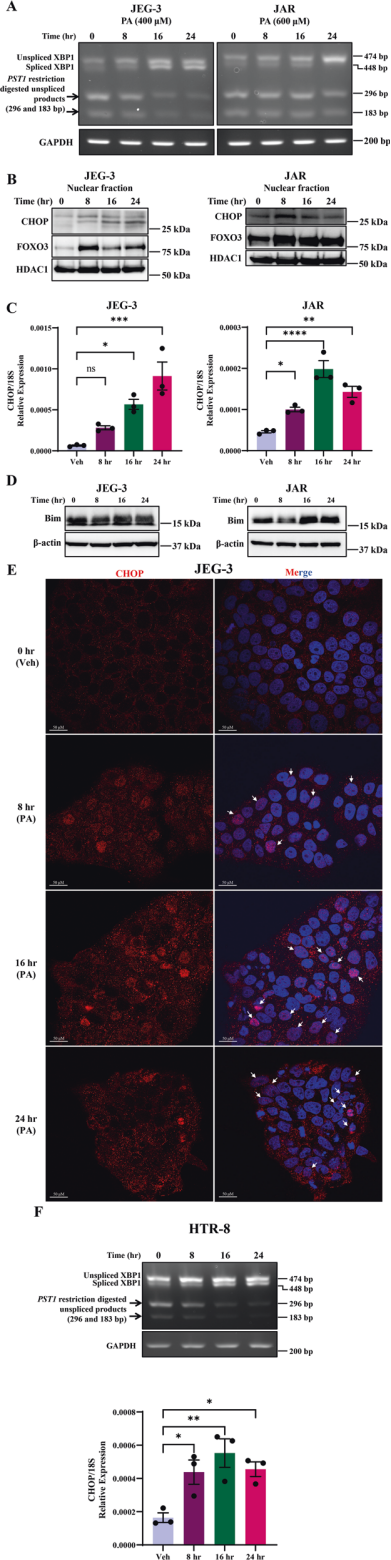
Palmitate activates XBP1 splicing and nuclear activation of CHOP and FoxO3 in trophoblasts. JEG-3 and JAR cells were treated with 400 µM of palmitate (PA) for 8, 16 and 24 h showed: **A** increased XBP splicing; **B** increased nuclear translocation of CHOP and forkhead family of transcription factor class O3 (FoxO3) after 8–24 h; **C** increased CHOP mRNA levels after 8–24 h of PA treatment relative to 18S rRNA; **D** increased levels of FoxO3a downstream targets: BIM and PUMA after 16–24 h in JAR cells; and **E** immunofluorescence staining of CHOP in JEG-3 cells with 8–24 h of 400 µM palmitate treatment showed enhanced CHOP nuclear localization compared to vehicle-treated cells. DAPI is used for nuclear staining. Merge of CHOP and DAPI showed increased colocalization of both staining (purple color). **F** Treatment of HTR-8 cells with 400 µM of PA for 8–24 h showed increased XBP1 splicing and decreased unspliced digested products and upregulation of CHOP mRNA expression compared to vehicle-treated cells. The images are representative images. Data presented as mean ± SEM, *n* = 3. *P < 0.05, **p < 0.01, ***p < 0.001, ****p < 0.0001 compared to vehicle-treated cells or PA-treated cells.

Another arm of ER stress is XBP1 mRNA splicing by ATF6 and IRE1α activation and the spliced XBP1 is a highly active transcription factor and potent inducer of Bip expression. We observed an increase in the spliced form of *XBP1* mRNA with the treatment of palmitate 400 µM after 16 and 24 h in placental trophoblasts compared to vehicle-treated cells (Fig. 4A). Further, we observed a decrease in unspliced XBP1 mRNA (474 bp) and decreased *PST1* restriction digested products of unspliced XBP1 mRNA with increasing time points of palmitate treatment in both JEG-3 and JAR cells (Fig. 4A). These data further support the activation of IRE1α and ER stress during trophoblast lipoapoptosis.

All three arms of ER stress (PERK, ATF6 and IRE1α) are known to activate the expression of CHOP, in particular spliced XBP1 protein can translocate to the nucleus as a transcription factor and induces CHOP expression. We sought to test the activation and role for CHOP during palmitate-induced trophoblast lipoapoptosis. Treatment of PA increased the nuclear levels of CHOP compared to vehicle-treated cells (Fig. 4B). Treatment of PA at 400 µM for 8–24 h to trophoblasts also increased the expression of CHOP mRNA transcripts (Fig. 4B, C). We also observed increased CHOP mRNA expression and increased spliced XBP1 levels after 8–24 h of PA in HTR-8 cells (Fig. 4F) In addition, we also tested the nuclear activation of another pro-apoptotic transcription factor, FoxO3 in trophoblast with palmitate treatment. Similar to CHOP, FoxO3 nuclear translocation was observed in the PA-treated trophoblast compared to vehicle-treated JEG-3 or JAR cells (Fig. 4B). The pro-apoptotic transcription factors like CHOP and FoxO3 can together induce the expression of their downstream target; BIM, a pro-apoptotic Bcl2-family protein. Treatment of PA to JEG-3 and JAR cells showed increased expression of BIM after 16–24 h compared to vehicle-treated cells. Actin levels were unchanged in palmitate and vehicle-treated cells (Fig. 4D). We also observed increased levels of BIM and PUMA expression with treatment of PA, 16–24 h in HTR-8 cells compared to actin as control loading (Fig. S2). Next, to validate the nuclear translocation of CHOP with the treatment of PA, we employed immunofluorescence analysis of CHOP in PA-treated cells and indeed observed increased nuclear levels of CHOP. The colocalization of nuclear staining (DAPI) and CHOP was observed after 8–24 h of palmitate treatment in JEG-3 cells (Fig. 4E). CHOP colocalization with DAPI is dramatically increased after 16 h of palmitate treatment compared to 8h-, 24h-time points and vehicle-treated cells (Fig. 4E), further supporting the role of CHOP in PA-induced trophoblast lipoapoptosis. These data suggest that PA induces a critical mediator of ER stress and results in splicing of *XBP1* mRNA which can activate the expression of CHOP and its nuclear translocation for its pro-apoptotic transcription factor function and induces trophoblast lipoapoptosis.

### Palmitate induces MAPKs activation in trophoblasts

**Fig. 5:**
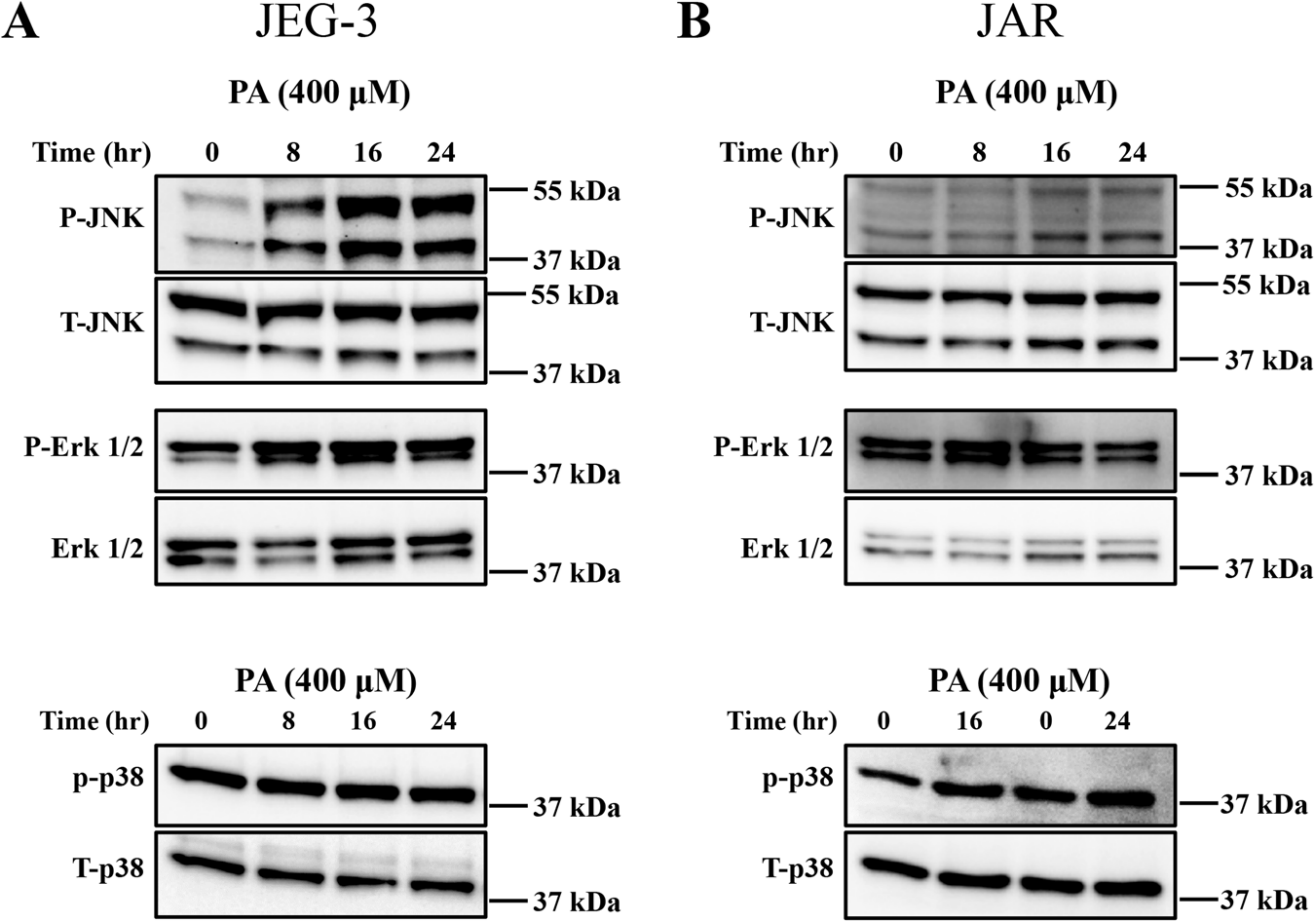
Palmitate induces MAPKs activation in trophoblasts. Cells were treated with 400 μM of palmitate (PA) and protein were collected at different time post treatment. Immunoblot analysis of MAPK activation via phosphorylation were observed with treatment of 400 µM of PA, 8–24 h in JEG-3 cells and showed increased phosphorylated forms of JNK and extracellular regulated kinase (ERK) 1/2, compared to the vehicle-treated cells. We did not observe any changes in the phospho-p38 MAPK with treatment of palmitate (400 µM, 8–24 h). Total JNK, total p38 MAPK and total ERK1/2 levels were unaltered between palmitate and vehicle treatment in both JEG-3 and JAR cells.

MAPKs, also commonly known as stress activated protein kinases (SAPKs) are a group of proteins known to be crucial to many complex cellular pathways including apoptosis. To examine the role of MAPKs, we treated trophoblasts with palmitate for different times (0 to 24 h) and tested the activation of c-Jun N-terminal kinase (JNK), p38 MAPK and extracellular signal-regulated kinase (ERK) via phosphorylation. Treatment of PA to both JEG-3 and JAR cells induces phosphorylation of both p54 and p46 isoforms of JNK in trophoblasts starting at 8 h post treatment and remained highly phosphorylated until 24 h, while total JNK levels remained the same at all time points tested (Fig. 5A, B). We also observed increased phosphorylation of ERK1/2 (p44/42) in both JEG-3 (Fig. 5A) and JAR cells (Fig. 5B); where JEG-3 cells showed sustained activation of ERK1/2 until 24 h. However, in JAR cells, activation of ERK1/2 level was only observed after 8–16 h of palmitate compared baseline (0 h) (Fig. 5A, B). Next, we analyzed the activation of p38 MAPK with the treatment of PA for 16–24 h and did not observe any changes in the levels of phosphorylated and total p38 MAPK (Fig. 5A). These data suggest that PA induces the activation of JNK and ERK in trophoblasts but did not activate p38 MAPK.

### Palmitate induces JNK-dependent trophoblast lipoapoptosis

**Fig. 6:**
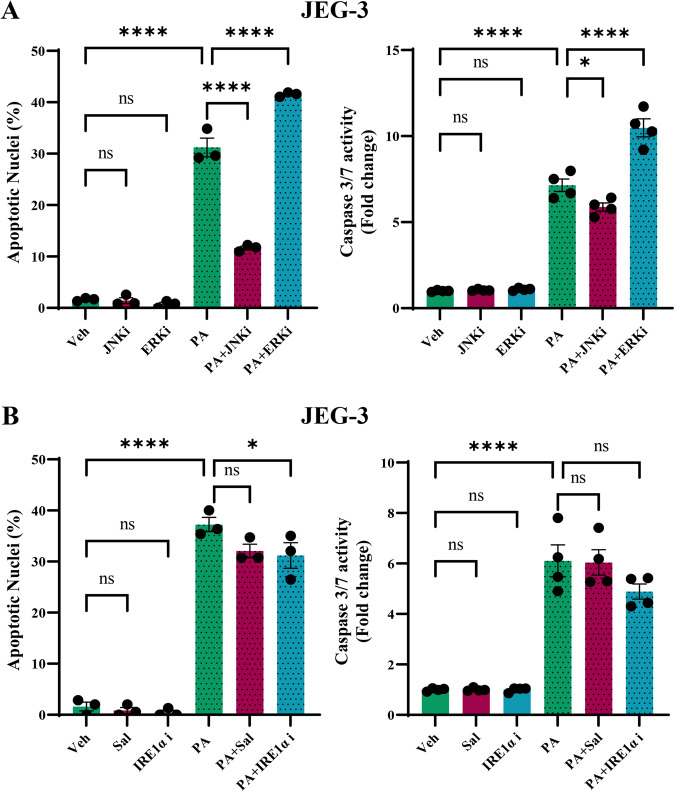
Palmitate-induced trophoblast lipoapoptosis is dependent on JNK activation. JEG-3 cells were treated with 400 μM of palmitate (PA) for a period of 24 h and apoptosis were analyzed with the treatment of JNK inhibitor (JNKi), ERK inhibitor (ERKi) (**A**). Cells were treated with PA, Salubrinal (Sal), IRE1α inhibitor (IRE1αi) along and in combination with PA (**B**). Data presented as mean ± SEM, *n* = 4. *P < 0.05, **p < 0.01, ***p < 0.001, ****p < 0.0001 compared to vehicle (Veh) treated cells or PA-treated cells.

To determine the critical role of MAPKs and ER stress activation during trophoblast lipoapoptosis, we used small molecule inhibitors of stress kinase and ER stress mediators and assessed trophoblast lipoapoptosis along with the treatment of palmitate. Inhibition of both JNK using anthrapyrazoline (SP600125), an irreversible ATP competitive inhibitor significantly prevented palmitate-induced trophoblast lipoapoptosis. Treatment of JEG-3 cells with PA increased the percent apoptotic nuclei and caspase 3/7 activity and cotreatment of PA and JNK inhibitor (JNKi) significantly prevented the increased percent apoptotic nuclei levels and caspase 3/7 activation (Fig. 6A). However, treatment of cells with PA and ERK inhibitor (ERKi) exacerbated palmitate-induced trophoblast lipoapoptosis as evidenced by a significantly increased percent apoptotic nuclei and caspase 3/7 activity compared to PA alone treated trophoblasts (Fig. 6A). Treatment of JNKi or ERKi alone did not show apoptosis compared to vehicle (Veh) treated cells. Together these results suggest, JNK activation in response to palmitate treatment is critical for trophoblast lipoapoptosis.

### Palmitate-induced trophoblast lipoapoptosis do not involve eIF2α activation

We used small molecule inhibitor of eIF2α (Salubrinal, 20 µM), and IRE1α inhibitor (STF-083010, 20 µM) to test the critical mediator of PA-induced trophoblast lipoapoptosis. PA-induced increase in percent apoptotic nuclei and caspase 3/7 activation is similar to Fig. 1. Treatment of trophoblasts with eIF2α inhibitor did not significantly alter PA-induced percent apoptotic nuclei and caspase 3/7 activity (Fig. 6B). IRE1α inhibitor showed a significant decrease in percent apoptotic nuclei levels compared to PA alone treated trophoblasts, However, IRE1α inhibition showed a trend of decreased PA-induced caspase 3/7 activity but it was not statistically significant.

### Palmitate activates stress granule formation in trophoblasts

**Fig. 7:**
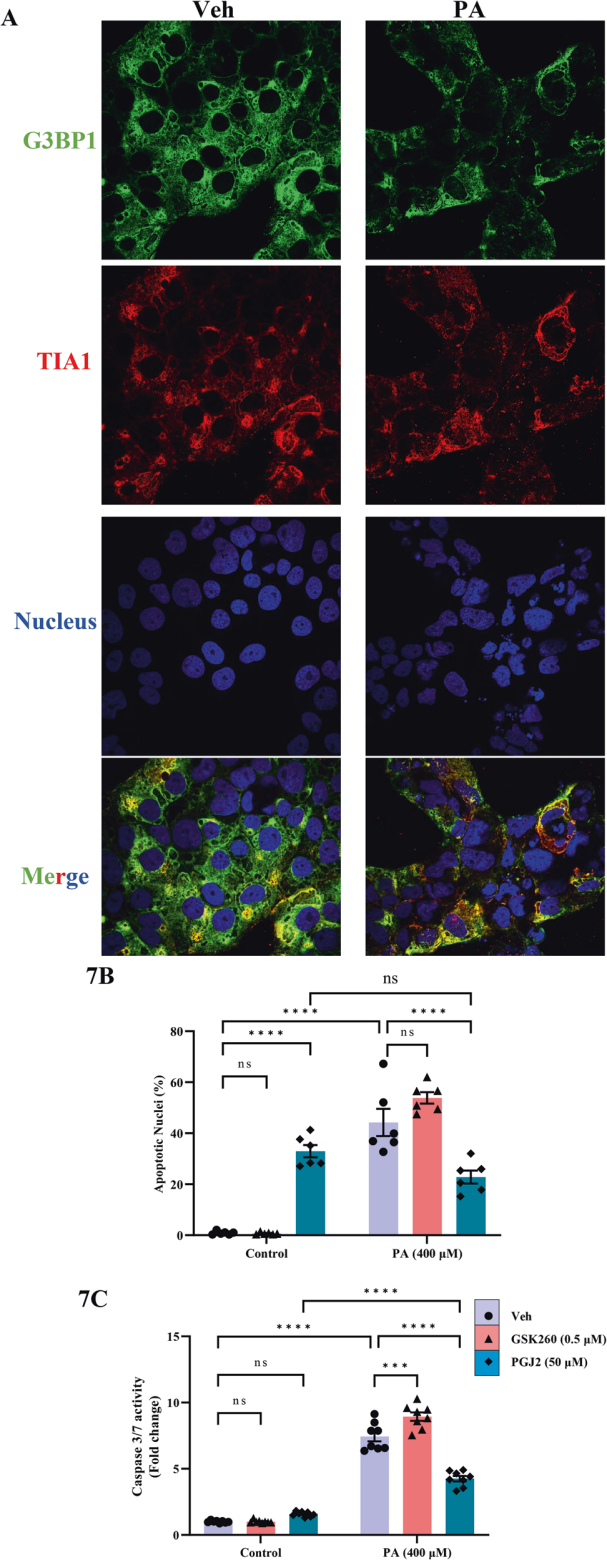
Palmitate activates stress granule formation in trophoblast. **A** JEG-3 cells were treated with 400 μM of palmitate (PA) for a period of 24 and the cells were stained with G3BP1 and TIA1 showed increased punctae in PA-treated cells compare to vehicle-treated cells. DAPI was used for nuclear staining. Merge of G3BP1 and TIA1 showed increased colocalization of both staining (purple color). The images are representative images. **B**, **C** Cells were treated with 400 μM of palmitate (PA) and with the cotreatment of PA and stress granule inhibitor (GSK260) and stress granule activator (PGJ2) for a period of 24 h and apoptosis was quantitated using nuclear morphological changes (**B**) and caspase 3/7 activity (**C**). Palmitate-induced trophoblast lipoapoptosis is blocked with the activation of stress granule formation using PGJ2. Data presented as mean ± SEM, *n* = 6. *P < 0.05, **p < 0.01, ***p < 0.001, ****p < 0.0001 compared to vehicle-treated cells or PA-treated cells.

Stress granules are liquid-liquid phase-separated assemblies of mRNAs, RNA binding proteins and translation initiator factors and are formed during various cellular stress [17]. Treatment of palmitate at 400 µM for 24 h dramatically increased the stress granule formation in placental trophoblasts as evidenced by the immunofluorescence staining of G3BP1 and TIA1, which are biomarkers of cellular stress granules (Fig. 7A). We did not observe the any changes in the protein levels of G3BP1 with the treatment of PA (Fig S3B). We also observed increased colocalization of ER resident protein, calnexin and TIA1 with the treatment of PA compared to vehicle-treated cells (Fig. S4).

### PA-induced trophoblast lipoapoptosis is prevented with the activation of stress granule formation

We next measured whether inhibition or activation of stress granule is critical for trophoblast lipoapoptosis using small molecules. GSK2606414 (GSK260), an inhibitor of PERK phosphorylation blocks phospho-eIF2α mediated stress granule formation (Stress granule inhibitor) and 15-DeoxyΔ^12,14^-prostaglandin J2 (15d-PGJ2) promotes eIF2α phosphorylation and activates integrated stress response (stress granule activator). GSK260 and PGJ2 were cotreated with PA and trophoblast apoptosis were measured. Inhibition of stress granule formation using GSK260 did not alter the levels of PA-induced percent apoptotic nuclei, however significantly increased PA-induced caspase 3/7 activity (Fig. 7B, C) suggesting that stress granule inhibition could potentially aggravate palmitate-induced apoptosis. In contrast, cotreatment of PA and PGJ2 resulted in significantly decreased PA-induced trophoblast lipoapoptosis as evidenced by a decrease in caspase activity and percent apoptotic nuclei compared to PA-alone treated cells. We also observed that PGJ2 by itself caused a significant increase in caspase-independent cell death (Fig. 7B, C). Together, these data suggest that stress granule formation with palmitate exposure could potentially be a cell survival strategy in placental trophoblasts.

## Discussion

**Fig. 8:**
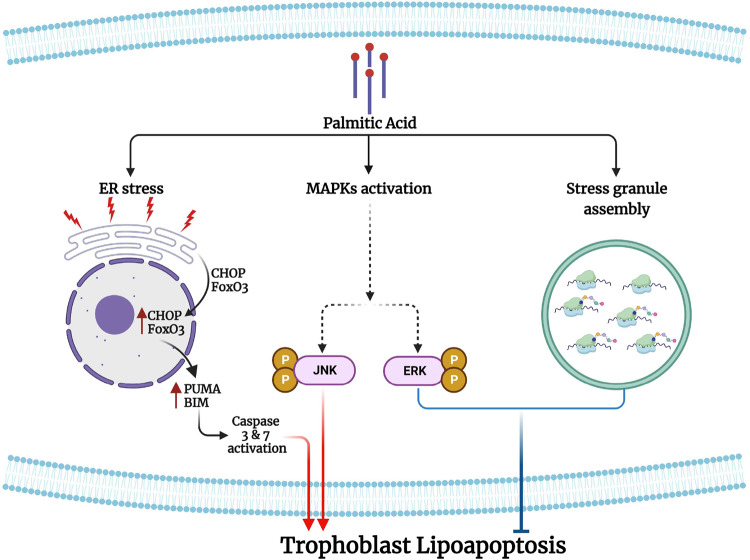
Schematic representation of PA-induced trophoblast lipoapoptosis. Palmitic acid (PA) induces integrated stress response pathways in trophoblast-like cells. In trophoblasts, PA treatment induces an increase in the phosphorylation of eIF2α and IRE1α, and cleavage of ATF-6 to its active form leading to the nuclear translocation of CHOP and FoxO3 nuclear translocation and induced the expression of their pro-apoptotic downstream targets like PUMA and BIM for lipoapoptosis. PA also activates MAPKs such as c-Jun N-terminal kinase (JNK) and ERK activation. Further, PA also induces stress granule assembly in trophoblasts. Activation of ERK and stress granule assembly shows a cell survival signal against PA-induced trophoblast lipoapoptosis. However, activation of JNK and caspases have a critical role on PA-induced trophoblast lipoapoptosis.

Saturated FFA, PA induces integrated stress response and lipoapoptosis to trophoblasts as evidenced by the activation of JNK, ER stress, caspases, CHOP and FoxO3. PA also induced cell survival signals like activation of ERK1/2 and granular stress signaling pathways. The schematic diagram (Fig. 8) represents the principal findings of this manuscript which are (1) PA induce a caspase-dependent apoptosis; (2) PA induces three arms of ER stress response; (3) nuclear translocation of CHOP and FoxO3 were observed in PA-induced trophoblast lipoapoptosis; (4) PA also induces the formation of stress granules; and (5) inhibition of JNK plays a critical role in PA-induced trophoblast lipoapoptosis; (6) inhibition of ERK and stress granules aggravated PA-induced trophoblast lipoapoptosis.

Maternal obesity is associated with lipotoxicity and decreased fatty acid oxidation in the placenta compared to non-obese mothers. We have earlier established that saturated FFAs induce a concentration-dependent increase in trophoblast lipoapoptosis [14]. We have also showed that co-treatment of palmitate with lipopolysaccharide exacerbates PA-induced trophoblast lipoapoptosis [14]. In the present study, we established that saturated FFAs induce a caspase-dependent lipoapoptosis in the trophoblasts. Palmitate is also known to induce placental macrophage lipoapoptosis and NLRP3 inflammasome activation, the latter can activate pro-caspase 1 to active caspase 1 [18]. The lipotoxic role of palmitate on macrophage-dependent inflammatory stimuli to trophoblasts during maternal obesity remains to be investigated. We show the activation of caspase 3 and 7 activity, however the role of other caspases like caspase-1 and 2 [19] during PA-induced trophoblast lipoapoptosis remained to be investigated.

Saturated free fatty acid, palmitate alters the morphology of ER and calnexin staining with distorted expression pattern. However, our data suggest that palmitate exposure to trophoblast does not alter the expression of calnexin, an ER resident protein that helps in glycoprotein protein folding. The flattened member vesicle found in ER is referred as cisternae or luminal space of ER. PA is known to induce expansion of cisternal ER and accumulation of phospholipids in the macrophages during ER stress. Other ER stress inducers like tunicamycin do not cause cisternal ER expansion. The cisternal ER expansion could be reflected as distorted calnexin expression pattern in PA-treated trophoblasts. Activation of XBP1 is known to increase the expression of choline cytidylyltransferase and choline phosphotransferase which has been shown to increase phosphatidyl choline synthesis and ER membrane expansion in adipocytes and macrophages. Further, PA has also been shown to induce dilatation of rough ER and compromise ER morphology and integrity [20]. However, the exact mechanism of PA-induced disrupted ER perinuclear morphological changes in the placental trophoblasts is unclear and will be determined in the future study.

Palmitate induces three arms of unfolded protein responses (PERK, IRE1α, and ATF6) and their downstream targets. We showed the activation of eIF2α via phosphorylation, a downstream target of PERK; increased phospho-IRE1α and spliced XBP1. We have also showed the activation of ATF-6 as evidenced by an increase in the levels of cleaved ATF-6 in trophoblasts. Activation of IRE1α could transcriptionally upregulate the expression of CHOP. As expected, CHOP expression is increased along with its nuclear translocation for the pro-apoptotic function including the transcription activation of BIM and PUMA expression. The timing of CHOP nuclear translocation with the increased PUMA and BIM expression were similar in palmitate-treated trophoblasts. The pro-apoptotic effect of CHOP is accompanied by the nuclear activation of FoxO3, another transcription factor [21], which can also induce the expression of BIM and PUMA in palmitate-induced cholangiocyte lipoapoptosis [13], suggesting that increased expression of PUMA could also be downstream mediator of CHOP and FoxO3 nuclear activation. Further, FoxO3 is known to induce the expression of pro-apoptotic microRNA 34a which can target protein deacetylase, sirtuin1, and anti-apoptotic proteins like KLF4 and cMET in cholangiocyte lipoapoptosis [14, 22]. Further studies are required to test the role and activation of microRNA 34a and its messenger RNA targets in PA-induced trophoblast lipoapoptosis.

Chronic ER stress results in the activation of stress kinases which can further enhance apoptosis. One such kinase is JNK and its activation via phosphorylation has been reported to be catalyzed by IRE1α. The stress kinase, JNK activation was found to be evident in all the time points tested and small molecule inhibition of JNK protected against palmitate-induced trophoblast lipoapoptosis. Activation of JNK was also reported in human placenta obtained from obese mothers compared to lean mothers [23]. However, inhibition of ER stress signaling pathway inhibitors did not protect against PA-induced trophoblast lipoapoptosis. JNK is a pro-apoptotic stress kinase and can be activated by IRE1α. Further, PUMA a downstream target of JNK is increased with palmitate exposure to trophoblasts and can contribute to trophoblast lipoapoptosis [24]. JNK specific phosphorylation of serine 473 residue of FoxO3 is known to induced pro-apoptotic function of FoxO3 and their nuclear translocation for lipoapoptosis [25]. Small molecule inhibition of IRE1α showed a small decrease with PA-induced trophoblast lipoapoptosis suggesting that the activation of JNK involves additional mediators. JNK can also be activated by TRAF/ASK1 dependent activation of MKK4/7 signaling pathway. JNK-dependent activation of its downstream signaling mediator, cJun a pro-apoptotic transcription factor is known to activate apoptosis [16] and their involvement needs to be studied in placental trophoblasts. The mechanism of JNK activation is complex and JNK can also be activated by GSK-3alpha and beta serine/threonine kinases [24, 26] and the role of JNK downstream target, cJun and FoxO3 activation and its interplay with PA-induced trophoblast lipoapoptosis are currently underway.

ERK1/2 signaling has been shown to protect ER stress-induced apoptosis and mostly viewed as cell survival signal [27]. In the present study, we also observed the activation of ERK as a cell survival signal, since small molecule inhibition of ERK1/2 significantly exacerbates PA-induced trophoblast lipoapoptosis. Further, chronic ER stress inhibits the activation of ERK and our data show that PA-induced increase in phospho-ERK1/2 levels were clearly observed in trophoblasts. Activated ERK1/2 is known to bind Bcl2 and can directly phosphorylate and exert its cell survival function [28]. Further, there was a decrease in the activation of ERK after 24 h of PA, which could result in loss of cell survival signal. Our data suggests that PA-induced ERK activation via phosphorylation is activating cell survival response to prevent trophoblast lipoapoptosis.

Palmitate-induced stress granules is another cell survival strategy that occurs in placental trophoblasts. Acute stress granule formation with the exposure of hypoxia, heat stress and sodium arsenite has been shown to inhibit pro-apoptotic signaling and promote cell survival [29]. Prevention of stress granule formation using genetic deletion of TIA1 prevents beta cell dysfunction [30]. Our data supports a previous publication proposing that formation of stress granules inhibits apoptosis by suppressing MAPK activation [29]. In contrast, PA-induced stress granules were also reported to be involved in pancreatic islet beta cell dysfunction. PA-induced stress granules formation recruit pancreatic and duodenal homeobox factor 1 (PDX1) and blocks the nuclear translocation of PDX1, thereby decreasing insulin secretion [30]. Despite the activation of cell survival signals like ERK and stress granule formation; palmitate still induced trophoblast lipoapoptosis. Inhibition of stress granules by using PERK inhibitor, GSK2606414 [31, 32] enhances PA-induced trophoblast lipoapoptosis. However, cotreatment of PGJ2 and palmitate inhibited PA-induced trophoblast lipoapoptosis. PGJ2 is an activator of stress granules, integrated stress response and peroxisomal proliferator activator gamma (PPARγ), respectively [33]. PGJ2 as a natural agonist of PPARγ could promote lipid droplets accumulation and increase the expression of stearoyl CoA desaturate 1 activity and has been shown to prevent PA-induced ER stress and JNK activation [34]. Interestingly, trophoblasts treated with only PGJ2 showed a caspase-independent cell death and the dual role of this anti-inflammatory prostaglandin in control and palmitate-treated trophoblasts remains to be investigated using an animal model of maternal obesity.

In conclusion, the saturated free fatty acid PA induces trophoblast lipoapoptosis and is mediated through activation of JNK. Palmitate also activates an integrated stress response; however small molecule inhibition of ER stress mediators does not significantly prevent palmitate-induced trophoblast lipoapoptosis. Further, inhibition of ERK aggravates PA-induced trophoblast lipoapoptosis, however stress granule activator prevents PA-induced lipoapoptosis. Further studies are required to develop a therapeutic target of JNK for the treatment of maternal obesity and prevent maternal obesity-induced trophoblast lipoapoptosis. Testing these signaling pathways in the human placenta of maternal obesity will be our future path of investigation.

### Supplementary information

Reproducibility checklist

Original Data File

Supplementary figures

## Data Availability

All the data generated or analyzed during this study are included in this published article and in supplementary information files.
