# Supplementary material for: Palmitate induces integrated stress response and lipoapoptosis in trophoblasts
Source: Cell Death Dis. 2024 Jan 11;15(1):31. doi: 10.1038/s41419-023-06415-6 (PMC10784287; doi:10.1038/s41419-023-06415-6)

Fig 1E

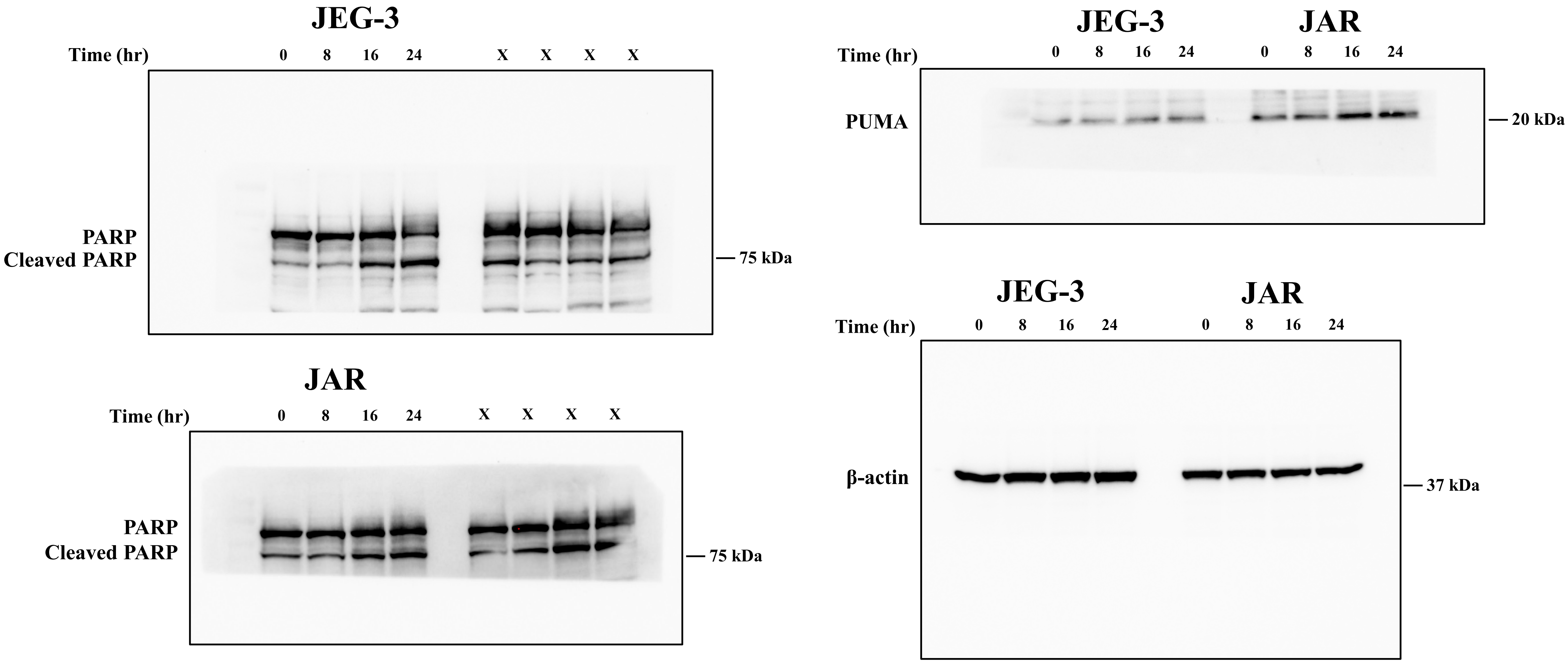
JEG-3
Time (hr)
0
8
16
24
X
X
X
X
PARP
Cleaved PARP
— 75 kDa
JAR
Time (hr)
0
8
16
24
X
X
X
X
PARP
Cleaved PARP
— 75 kDa
JEG-3
JAR
Time (hr)
0
8
16
24
0
8
16
24
PUMA
— 20 kDa
JEG-3
JAR
Time (hr)
0
8
16
24
0
8
16
24
β-actin
— 37 kDa

Fig 1F

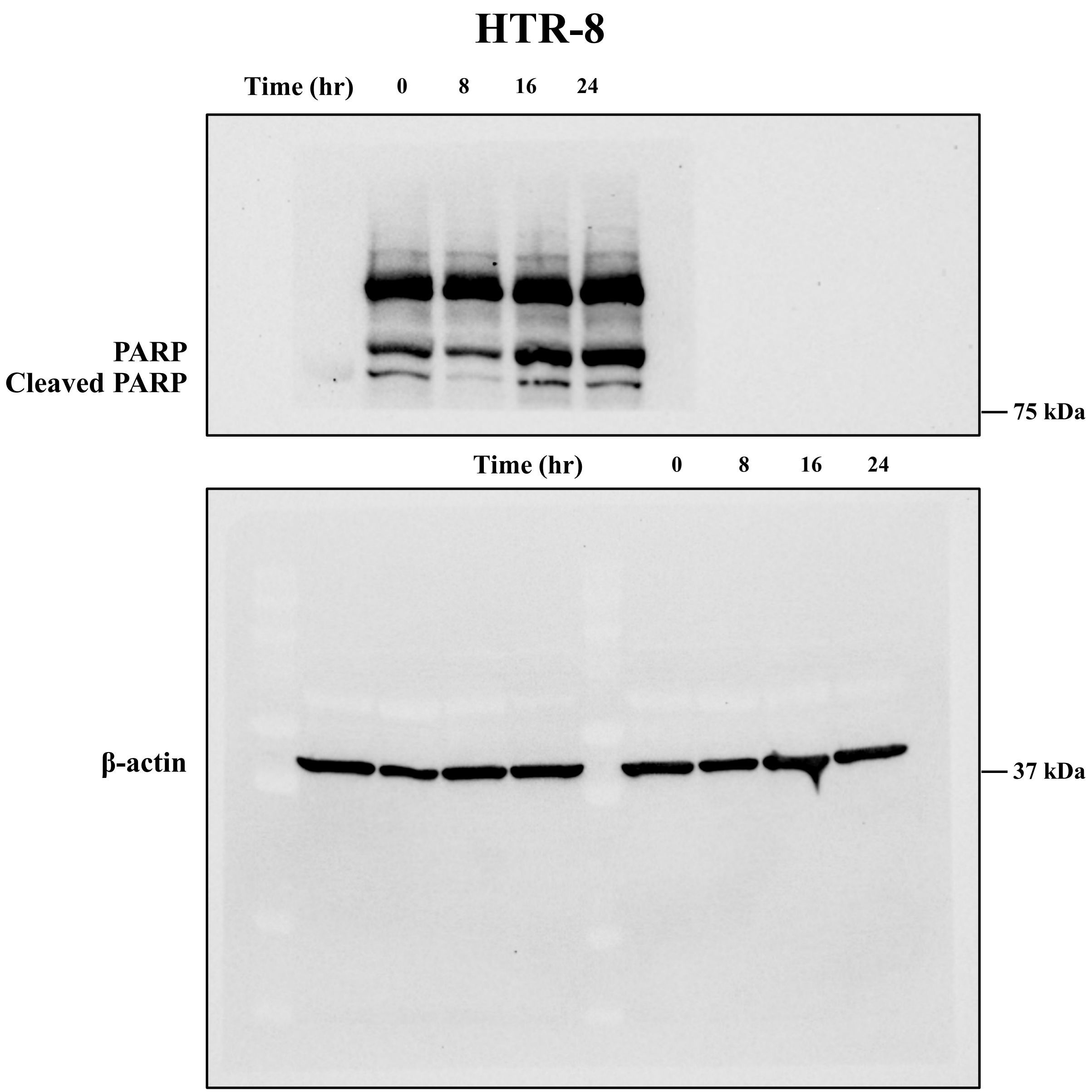
HTR-8
Time (hr) 0 8 16 24
PARP
Cleaved PARP
75 kDa
Time (hr) 0 8 16 24
β-actin
37 kDa

Fig 3C

JEG-3

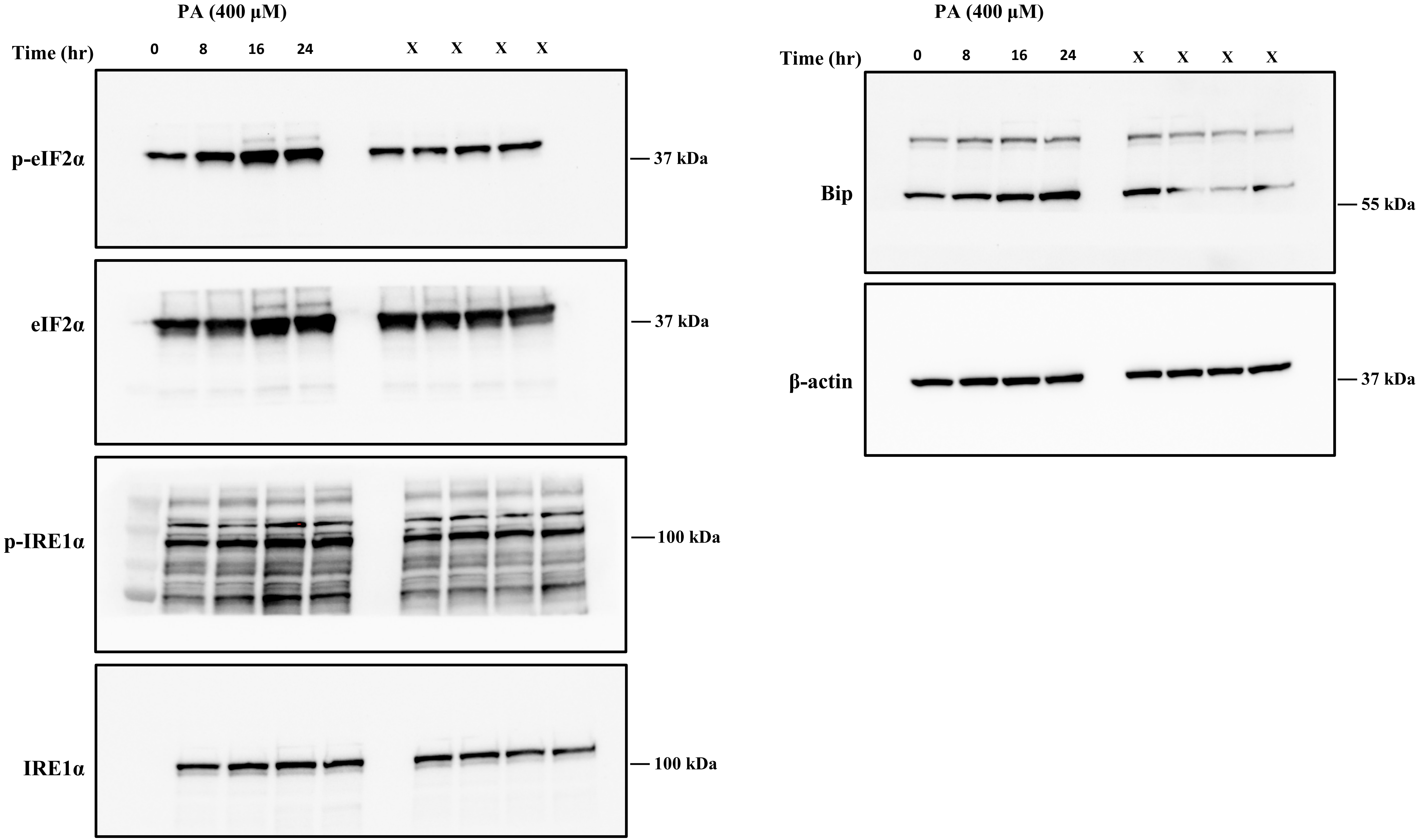
PA (400 µM)
Time (hr) 0 8 16 24 X X X X
p-eIF2α
37 kDa
eIF2α
37 kDa
p-IRE1α
100 kDa
IRE1α
100 kDa
PA (400 µM)
Time (hr) 0 8 16 24 X X X X
Bip
55 kDa
β-actin
37 kDa

Fig 3C

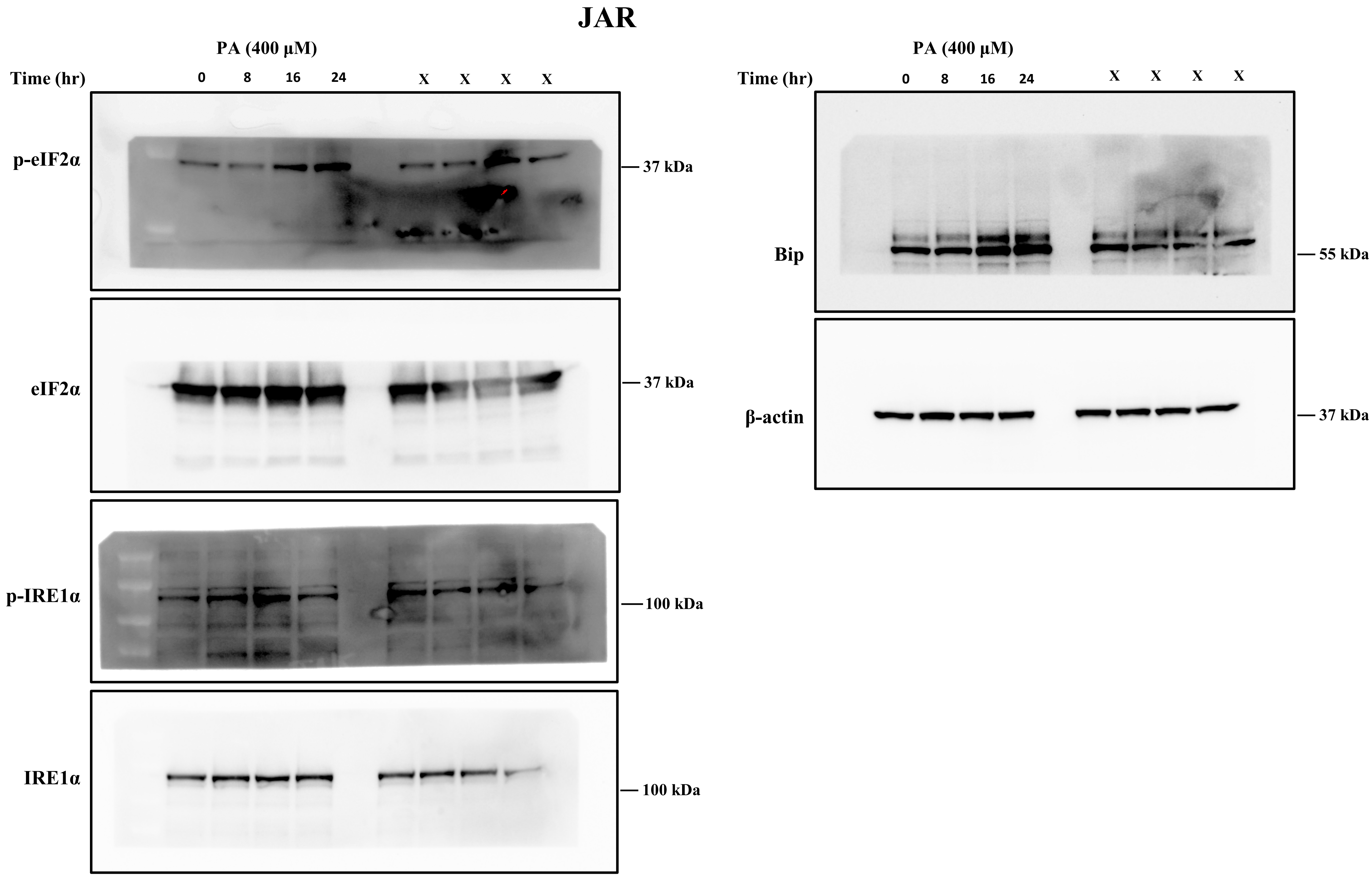
JAR
PA (400 µM)
Time (hr)
0
8
16
24
X
X
X
X
p-eIF2α
37 kDa
eIF2α
37 kDa
p-IRE1α
100 kDa
IRE1α
100 kDa
PA (400 µM)
Time (hr)
0
8
16
24
X
X
X
X
Bip
55 kDa
β-actin
37 kDa

Fig 3D

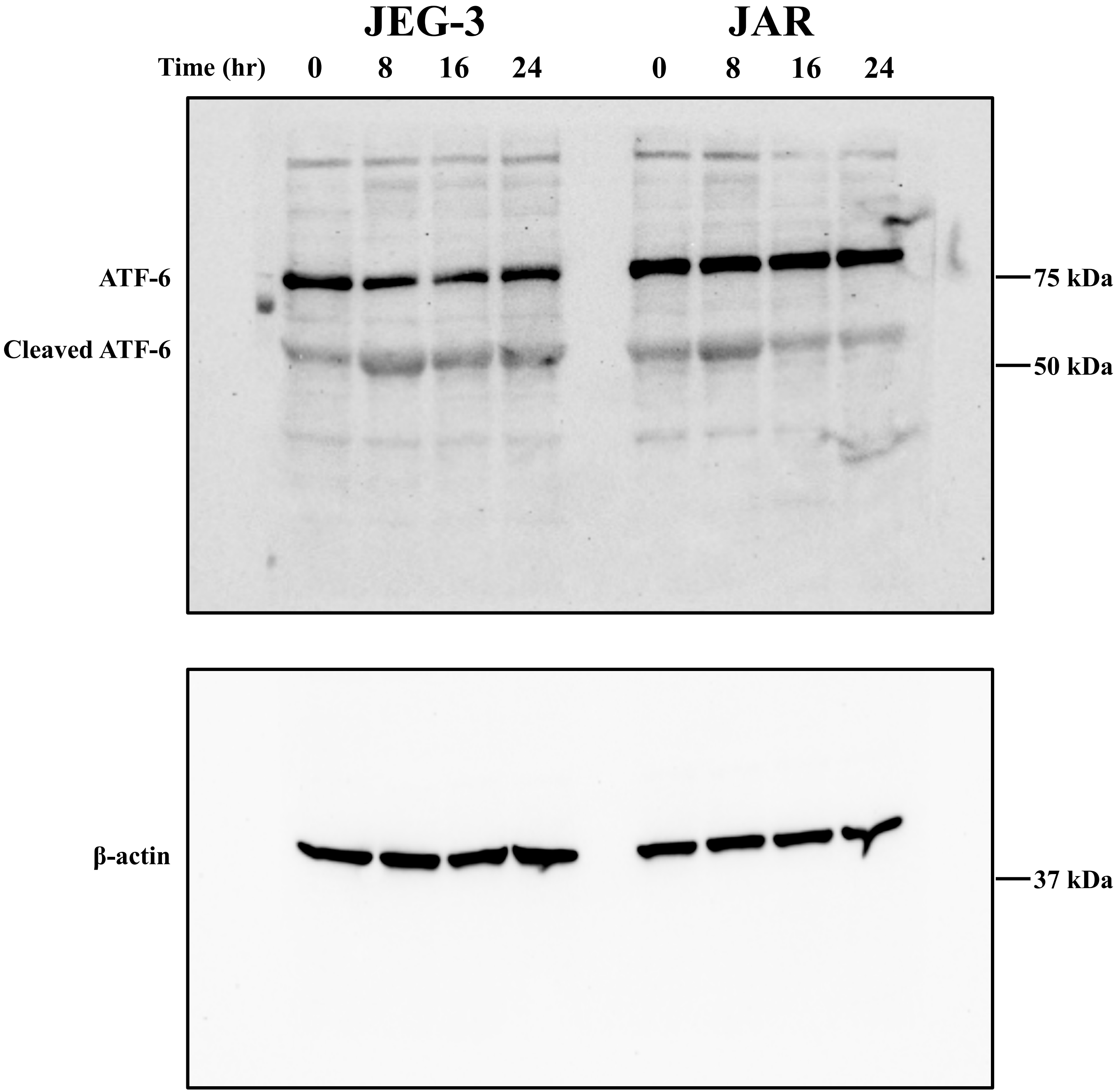

JEG-3
JAR
Time (hr)
0
8
16
24
0
8
16
24
ATF-6
Cleaved ATF-6
75 kDa
50 kDa
β-actin
37 kDa

Fig 3E

HTR-8

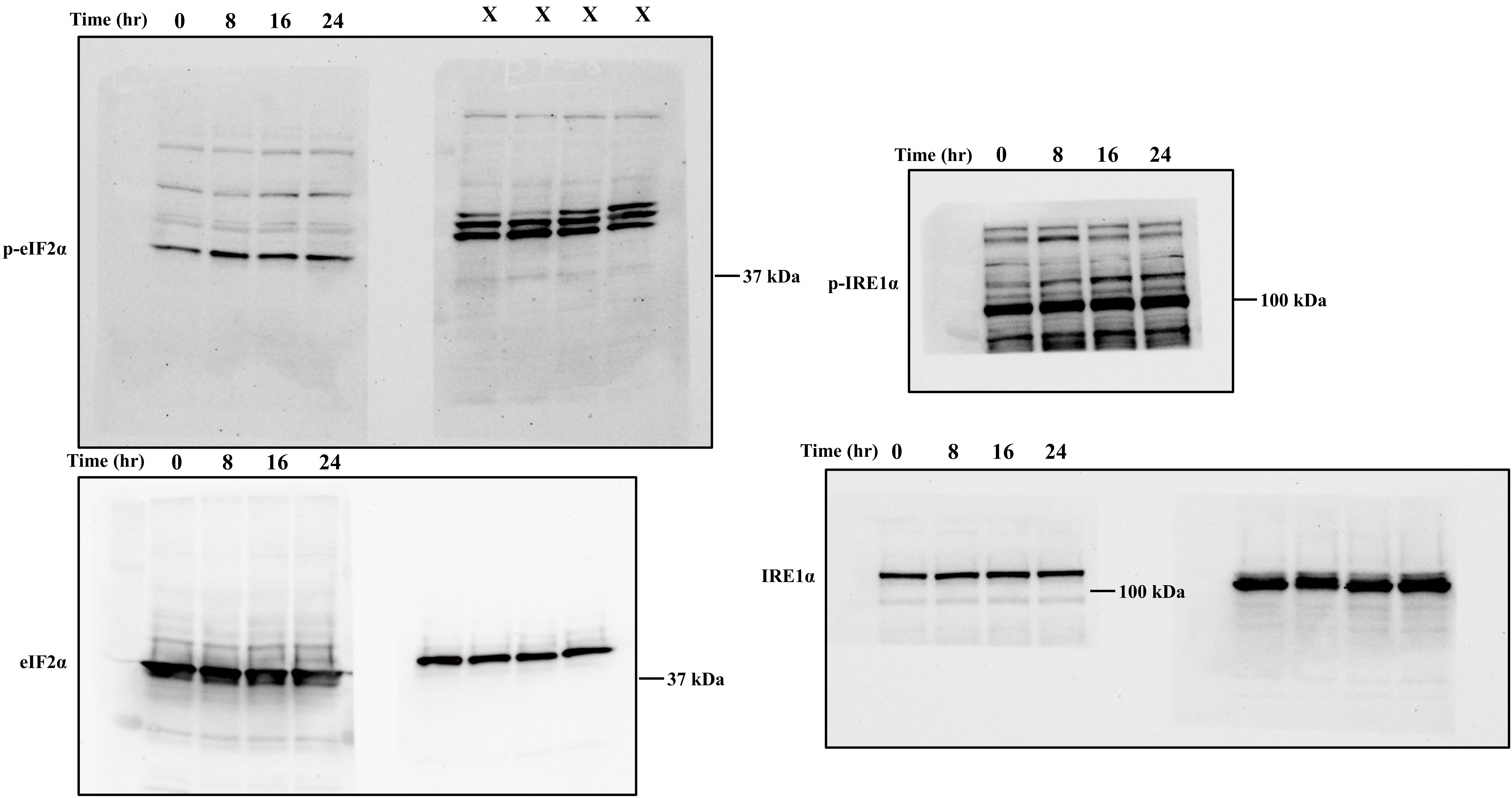
Time (hr) 0 8 16 24
X X X X
p-eIF2α
37 kDa
Time (hr) 0 8 16 24
p-IRE1α
100 kDa
Time (hr) 0 8 16 24
eIF2α
37 kDa
Time (hr) 0 8 16 24
IRE1α
100 kDa

Fig 3E

HTR-8

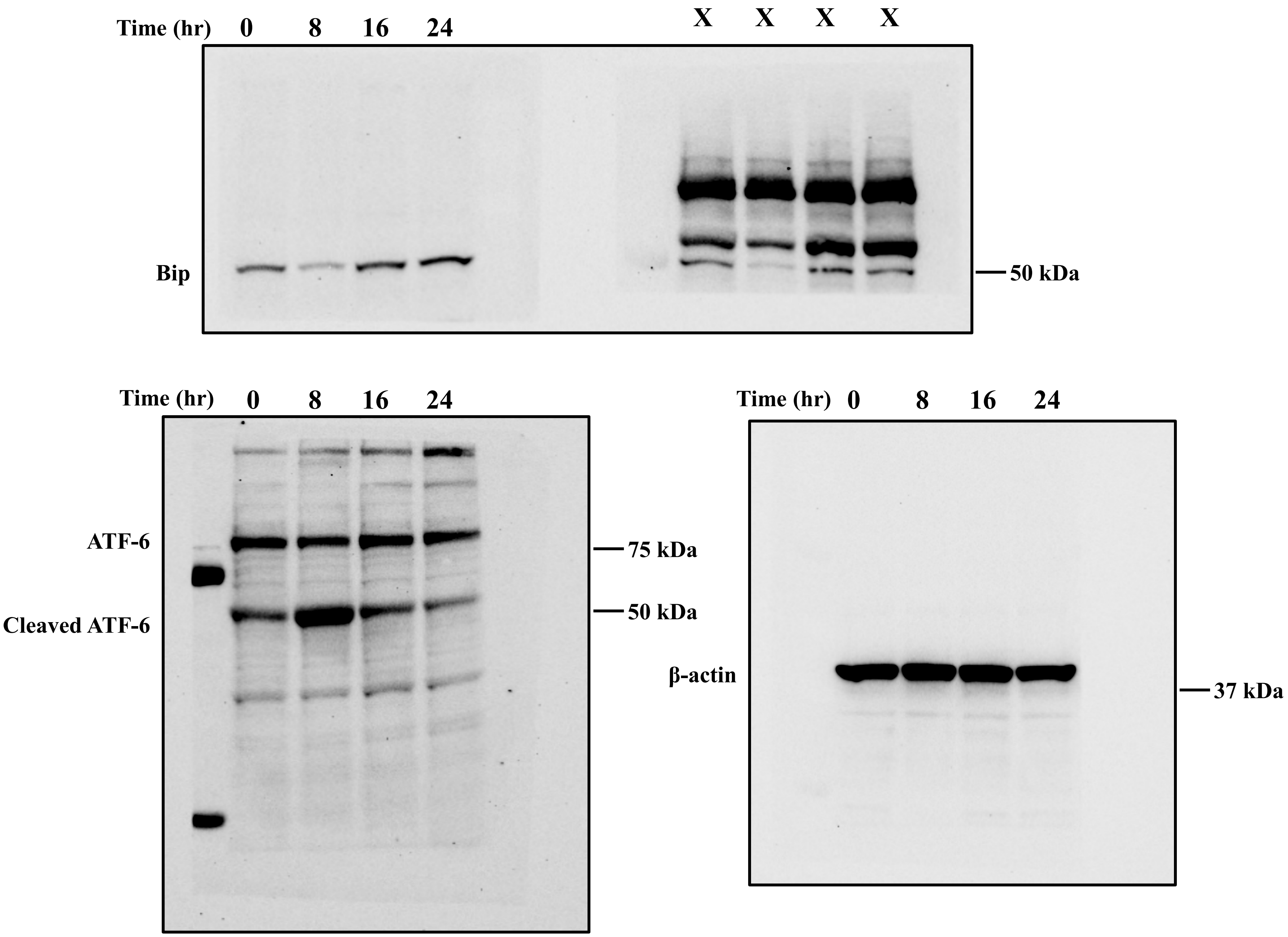
Time (hr)
0
8
16
24
X
X
X
X
Bip
50 kDa
Time (hr)
0
8
16
24
ATF-6
Cleaved ATF-6
75 kDa
50 kDa
Time (hr)
0
8
16
24
β-actin
37 kDa

Fig 4B

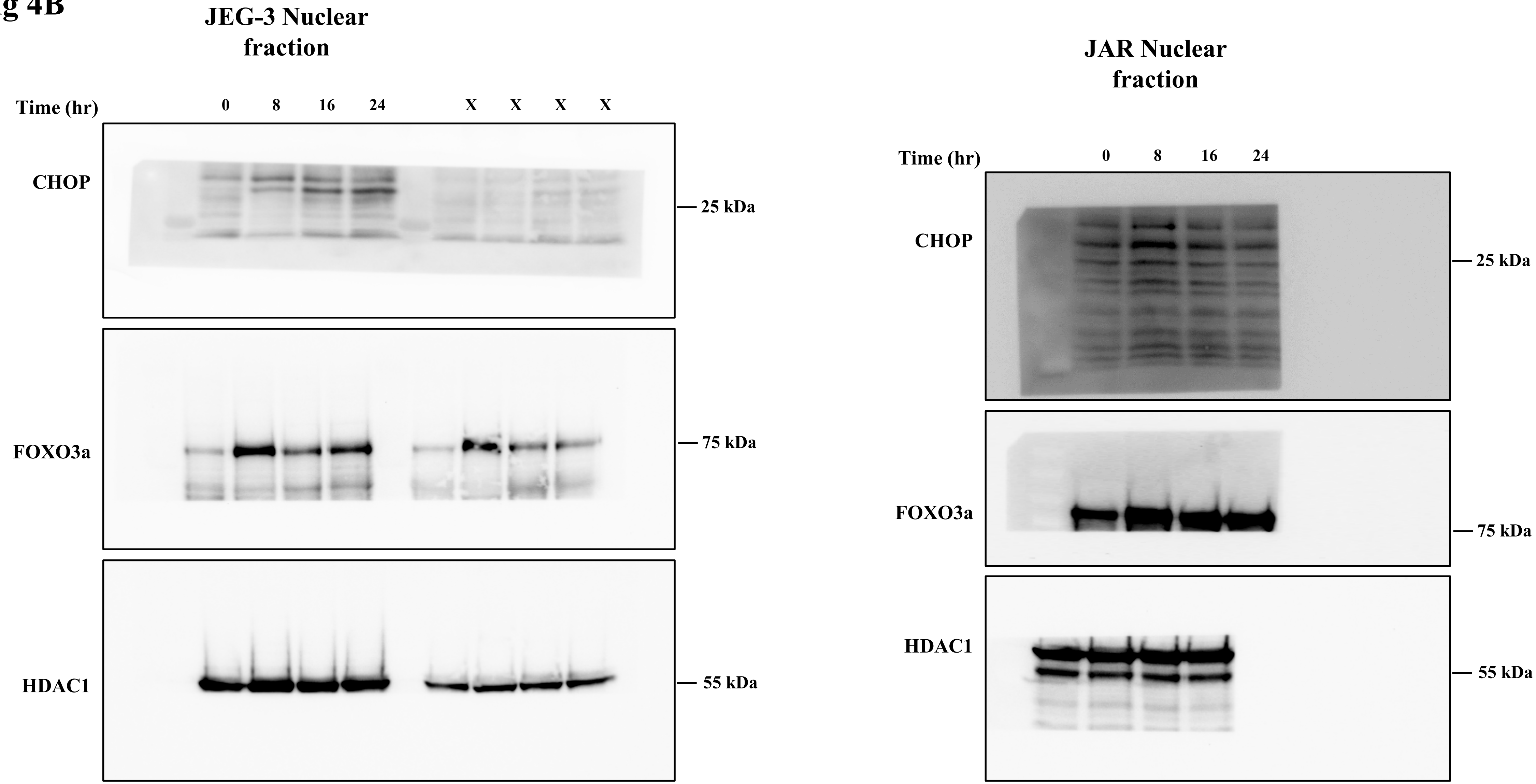
JEG-3 Nuclear fraction
Time (hr)
0
8
16
24
X
X
X
X
CHOP
25 kDa
FOXO3a
75 kDa
HDAC1
55 kDa
JAR Nuclear fraction
Time (hr)
0
8
16
24
CHOP
25 kDa
FOXO3a
75 kDa
HDAC1
55 kDa

Fig 4D

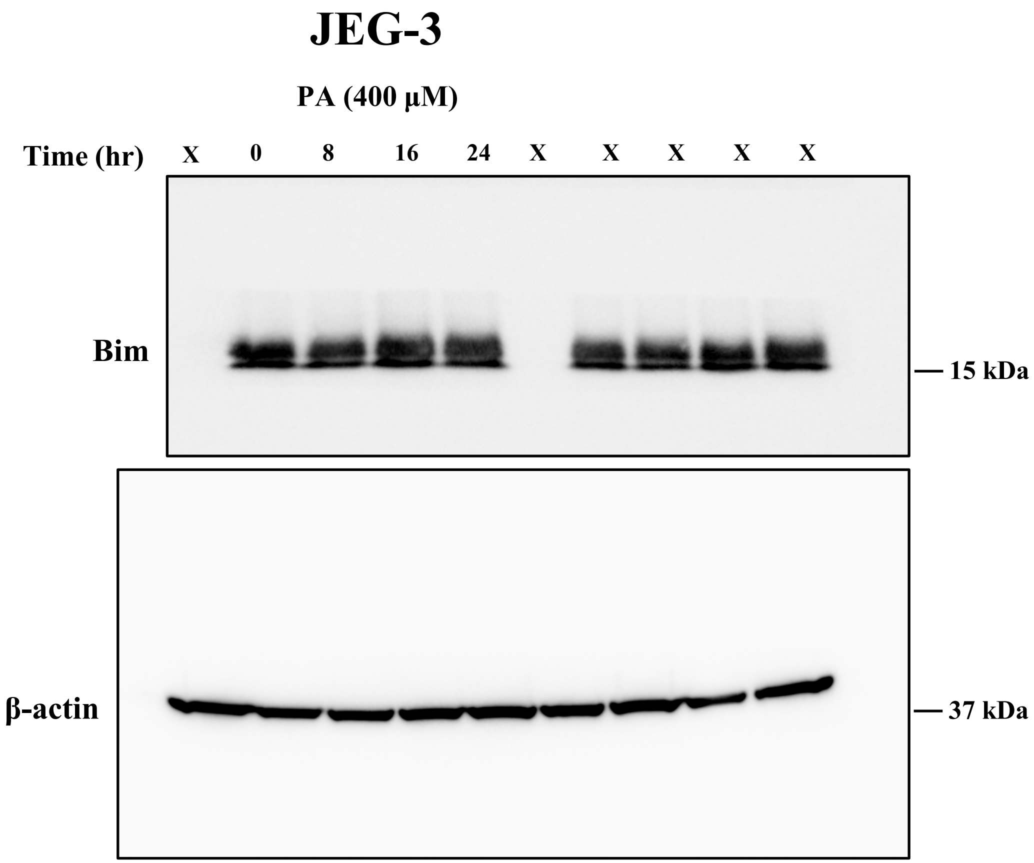

This b-actin has been used in Fig S3B again

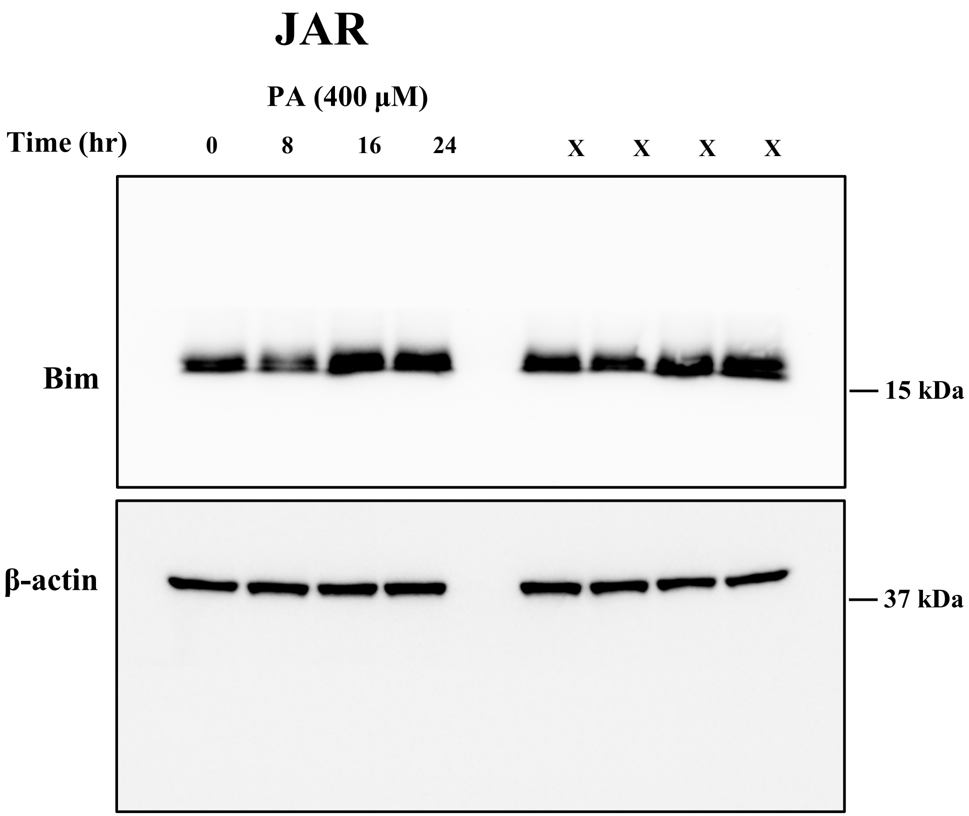

Fig 5A

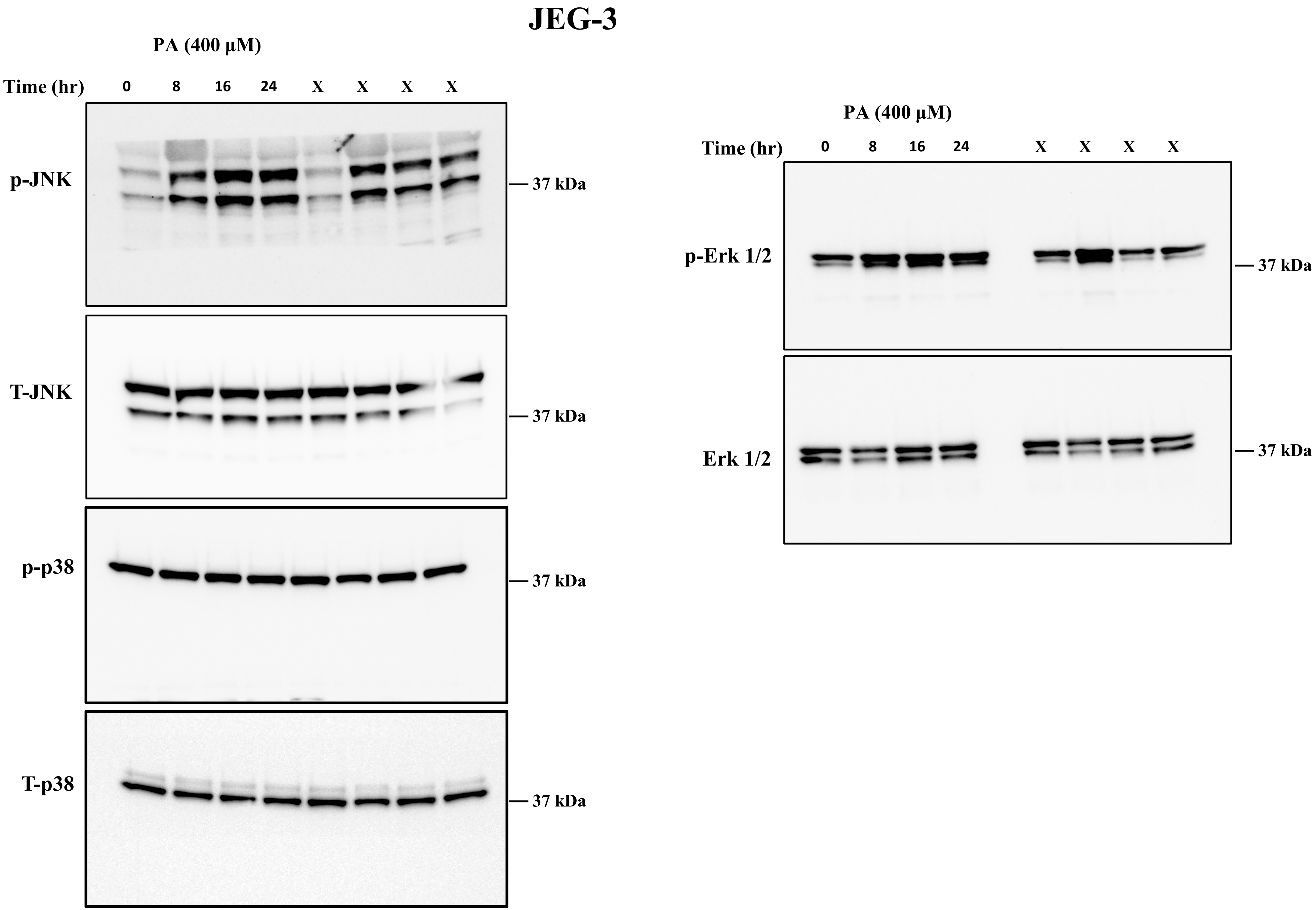
JEG-3
PA (400 µM)
Time (hr) 0 8 16 24 X X X X
p-JNK
37 kDa
T-JNK
37 kDa
p-p38
37 kDa
T-p38
37 kDa
PA (400 µM)
Time (hr) 0 8 16 24 X X X X
p-Erk 1/2
37 kDa
Erk 1/2
37 kDa

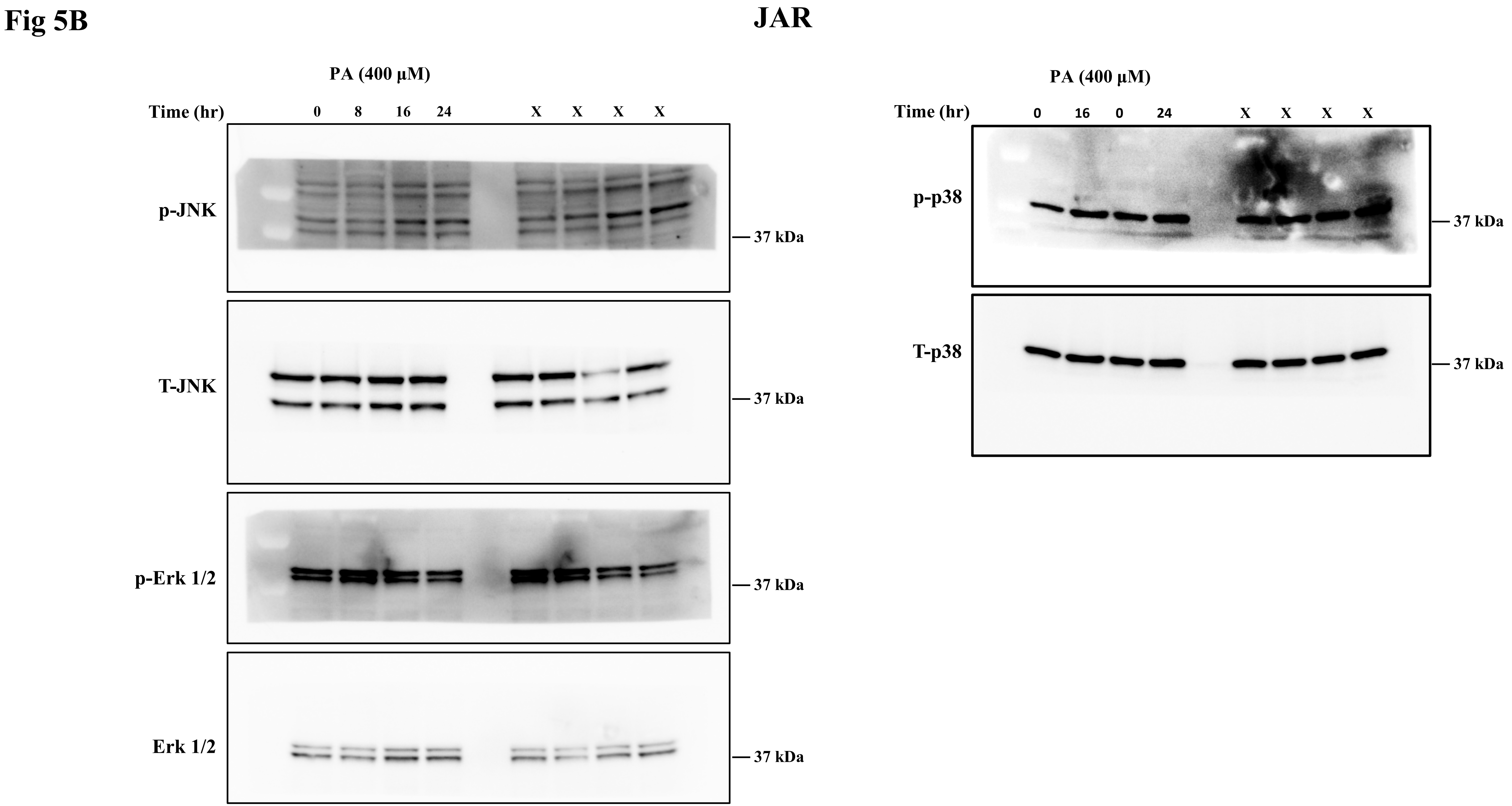
Fig 5B
JAR
PA (400 µM)
Time (hr)
0
8
16
24
X
X
X
X
p-JNK
37 kDa
T-JNK
37 kDa
p-Erk 1/2
37 kDa
Erk 1/2
37 kDa
PA (400 µM)
Time (hr)
0
16
0
24
X
X
X
X
p-p38
37 kDa
T-p38
37 kDa

Fig S2

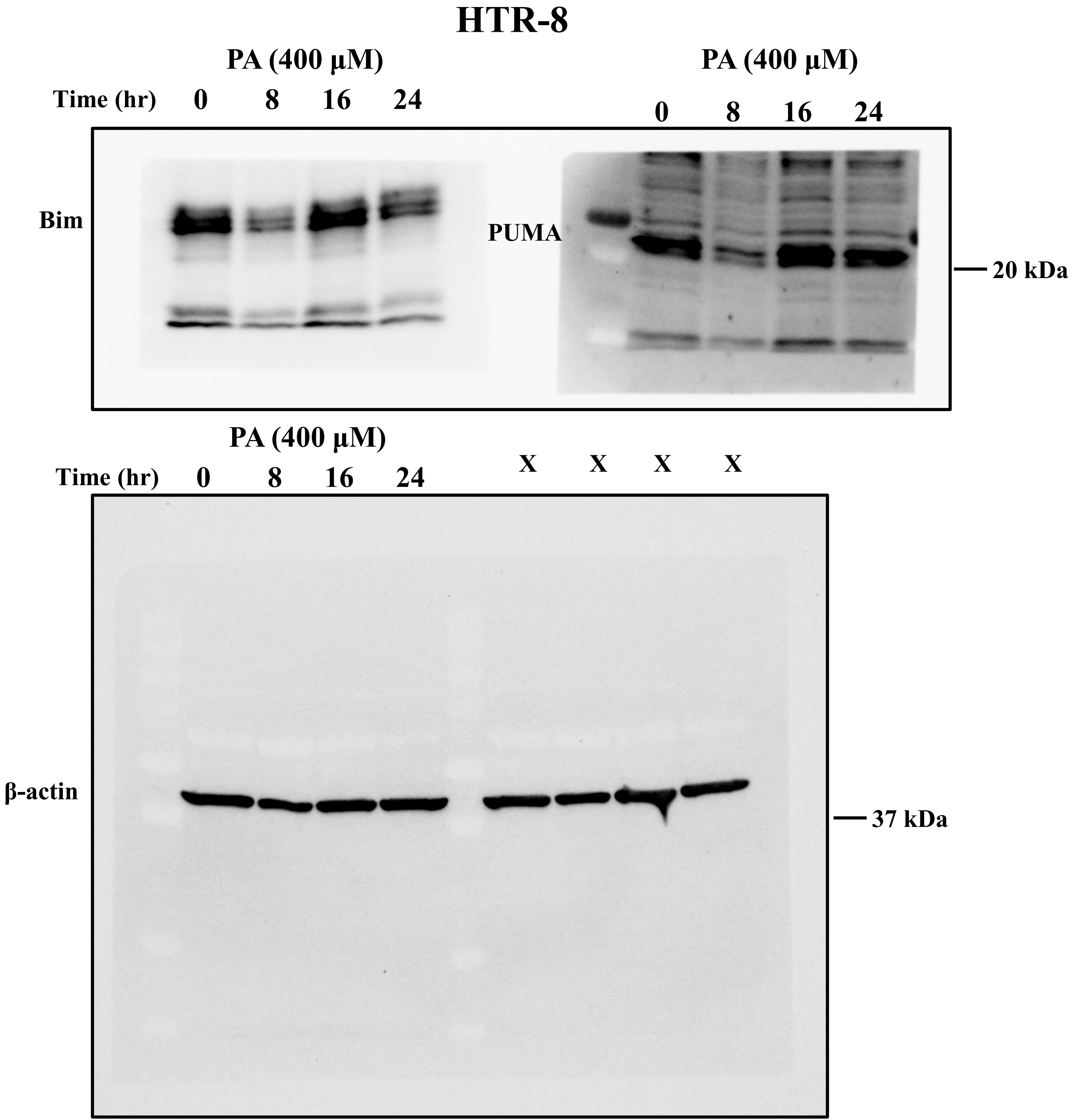
HTR-8
PA (400 µM)
Time (hr) 0 8 16 24
Bim
PA (400 µM)
0 8 16 24
PUMA
20 kDa
PA (400 µM)
Time (hr) 0 8 16 24
X X X X
β-actin
37 kDa

Fig S3 A

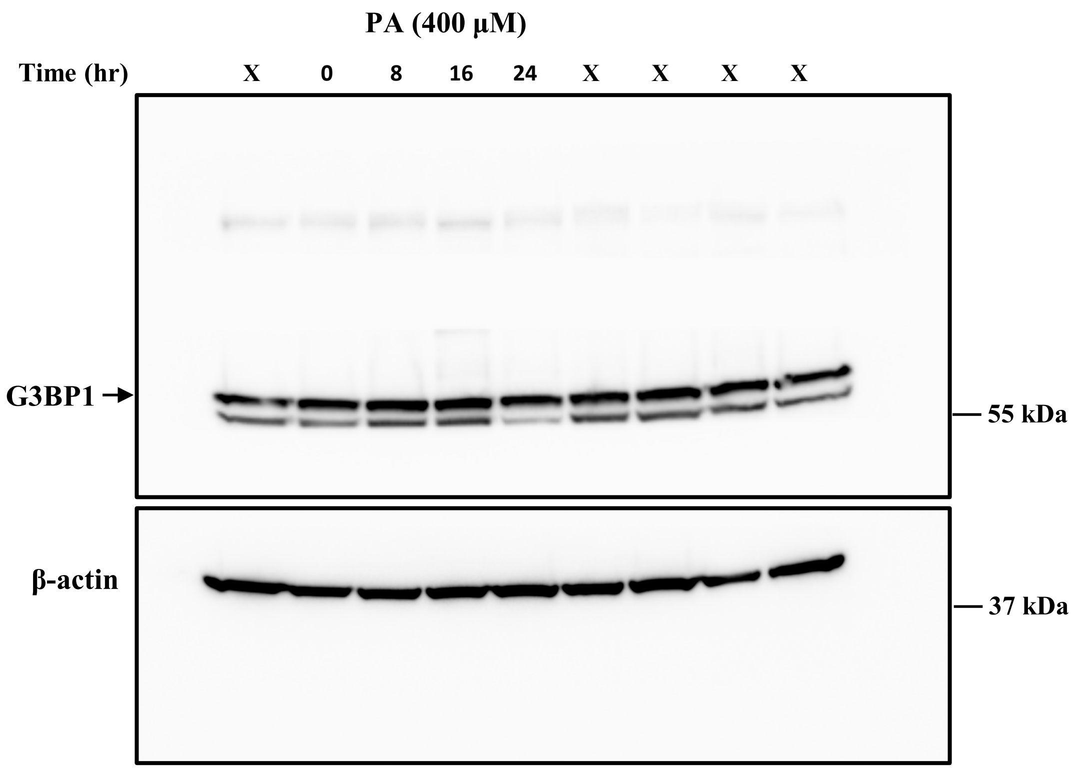

Fig S3 B

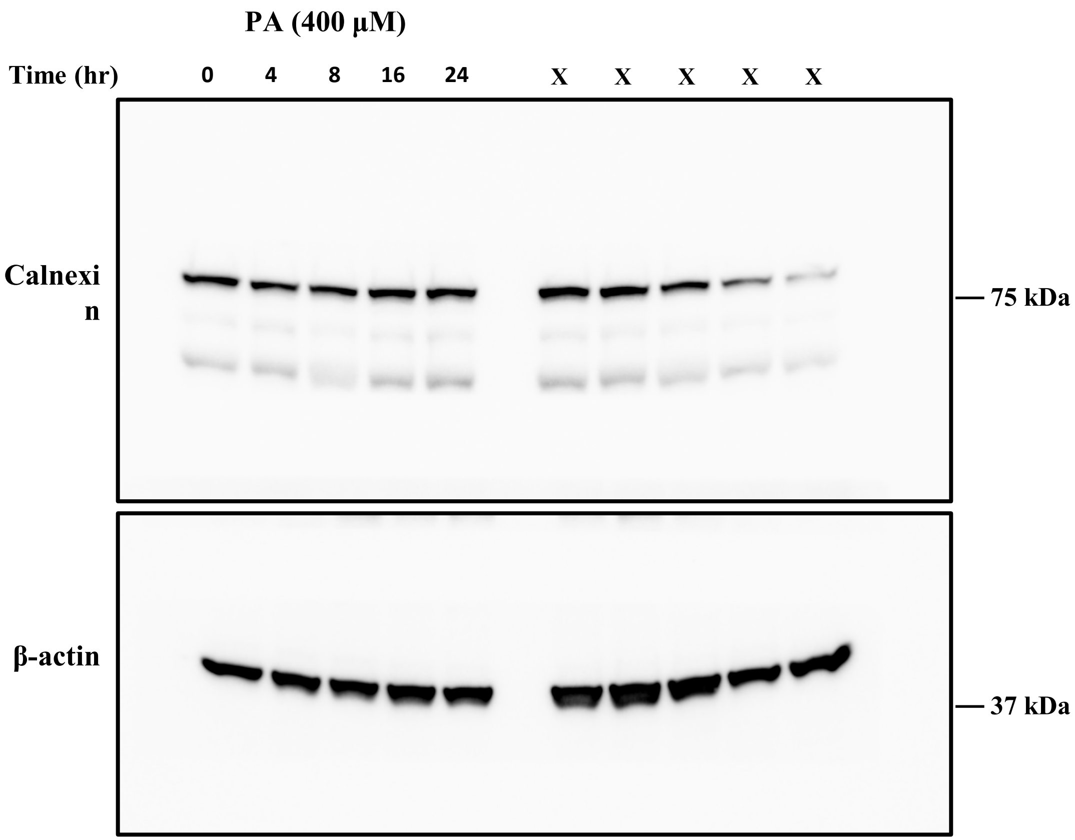

Supplement: Supplementary file 2 — Original Data File [file 41419_2023_6415_MOESM2_ESM.pdf]
