# Supplementary material for: Palmitate induces integrated stress response and lipoapoptosis in trophoblasts
Source: Cell Death Dis. 2024 Jan 11;15(1):31. doi: 10.1038/s41419-023-06415-6 (PMC10784287; doi:10.1038/s41419-023-06415-6)
Supplement: Supplementary file 3 — Supplementary figures [file 41419_2023_6415_MOESM3_ESM.docx]

**Palmitate induces Integrated Stress Response and Lipoapoptosis in Trophoblasts**

Prakash Kumar Sahoo^1^, Chandan Krishnamoorthy^1^, Jennifer R. Wood^2^, Corrine Hanson ^3^, Ann Anderson-Berry^4^, Justin L. Mott^5^, Sathish Kumar Natarajan^1,3^*

^1^Department of Nutrition and Health Sciences, University of Nebraska-Lincoln; ^2^ Department of Animal Sciences, University of Nebraska-Lincoln; ^3^College of Allied Health Professions Medical Nutrition Education, University of Nebraska Medical Center, Omaha, NE, ^4^Department of Pediatrics, University of Nebraska Medical Center, Omaha, NE. ^5^Department of Biochemistry and Molecular Biology, University of Nebraska Medical Center, Omaha, NE

**Running title: *Palmitate-induced trophoblast lipotoxicity***

*Address for Correspondence: Sathish Kumar Natarajan, PhD

Associate Professor, Department of Nutrition & Health Sciences

University of Nebraska-Lincoln

229 Filley Hall, Lincoln, NE 68583-0806

Tel: +1 402-472-3716

E-mail: [snatarajan2@unl.edu](mailto:snatarajan2@unl.edu)

ORCID: orcid.org/0000-0001-7491-8592

**Key words:** Maternal obesity, Endoplasmic reticulum stress, granular stress, free fatty acids, mitogen-activated protein kinase

**
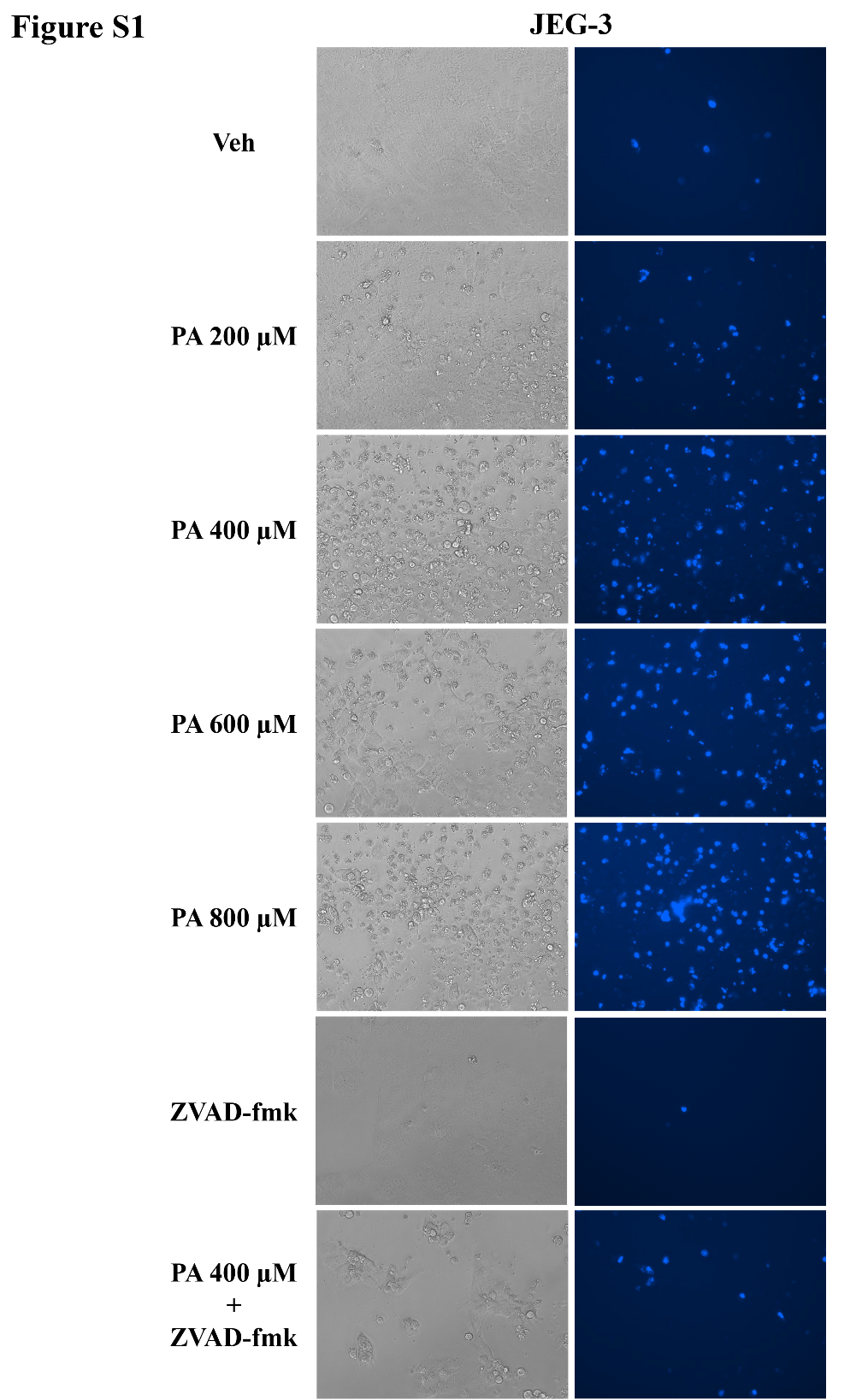
**

**Supplementary Figure 1. J**EG-3 cells were treated with either increasing concentration of PA or co-treated with Z-VAD-fmk (50 µM) for 24 h and apoptotic cells were analyzed using DAPI staining via EVOS FL fluorescence microscope. PA treated cells showed gradual increase in DAPI positive nuclei, while co-treatment of PA and Z-VAD-fmk showed reduced numbers of DAPI positive nuclei compared to PA alone treated cells. Treatment of vehicle (Veh) or Z-VAD-fmk alone did not induce any increase in DAPI positive cells. Images are representative from individual treatment groups.

**
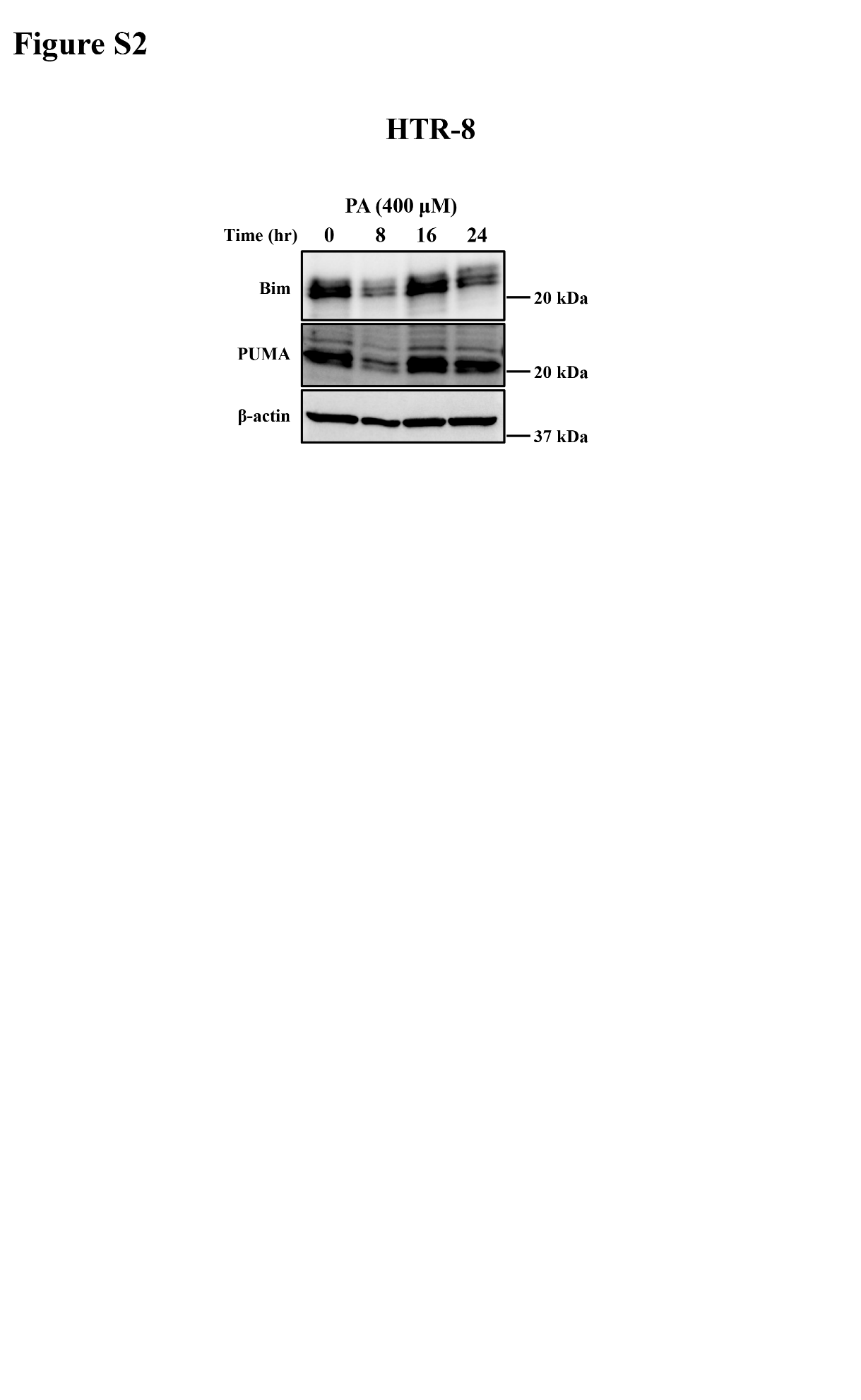
**

**Supplementary Fig S2:** HTR-8 cells were treated with PA (400 µM) for different times and analyzed using immunoblot. Pro-apoptosis mediator BIM and PUMA expression was subtly enhanced at 16 h post PA treatment.

**
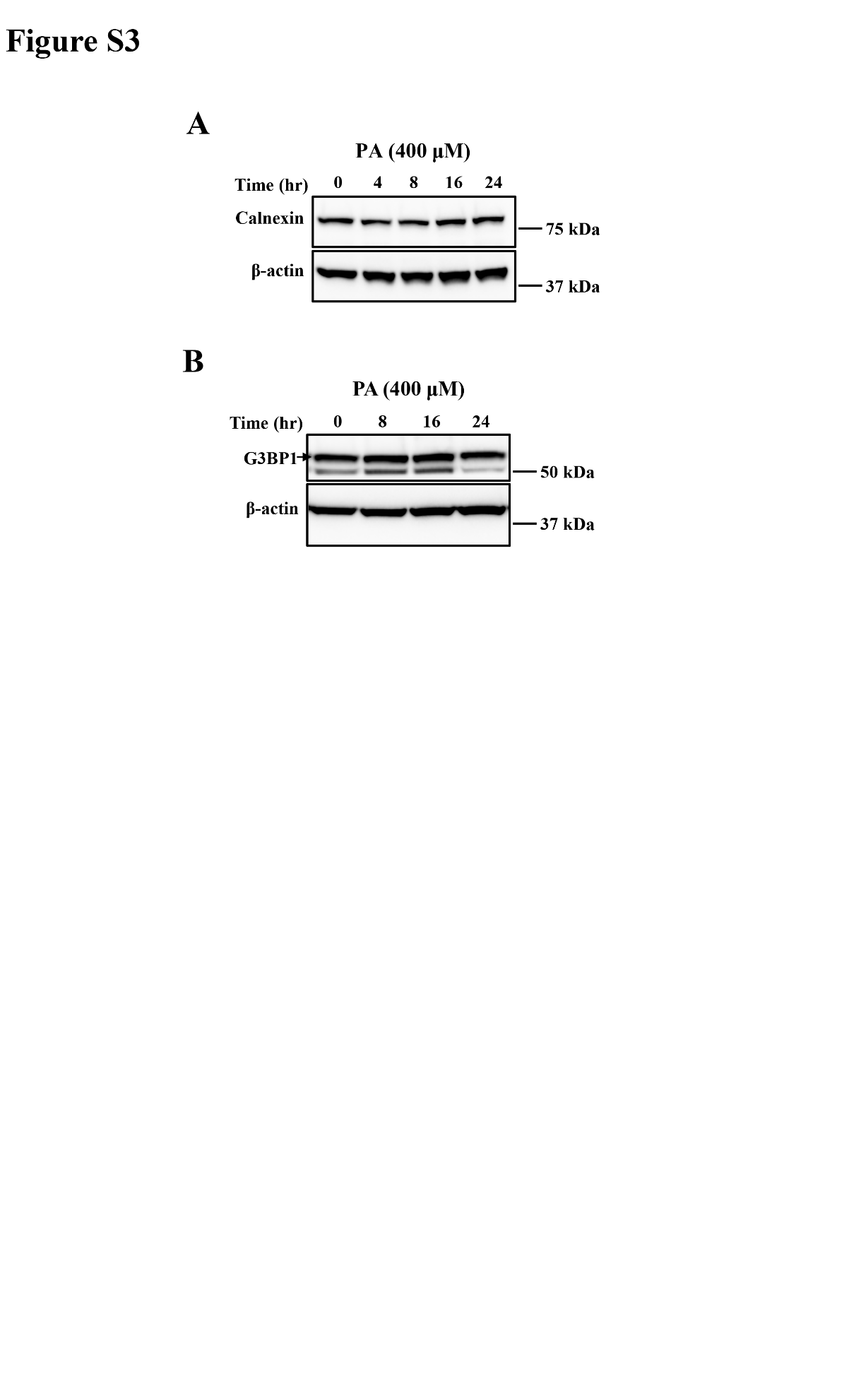
**

**Supplementary Fig S3:** **PA has no effect on Calnexin and G3BP1 protein level**. JEG-3 cells were treated with 400 μM of PA for a period of 24 hr and protein levels of Calnexin (A) and G3BP1 (B) were analyzed using Immunoblot analysis. We did not observe any changes in the protein levels of either Calnexin or G3BP1 at different times in PA treated cells compared to Veh treated cells. Beta-actin was used as loading control and remained same in all the treatment conditions. Beta actin used in figure S1B is also used in figure 4D.


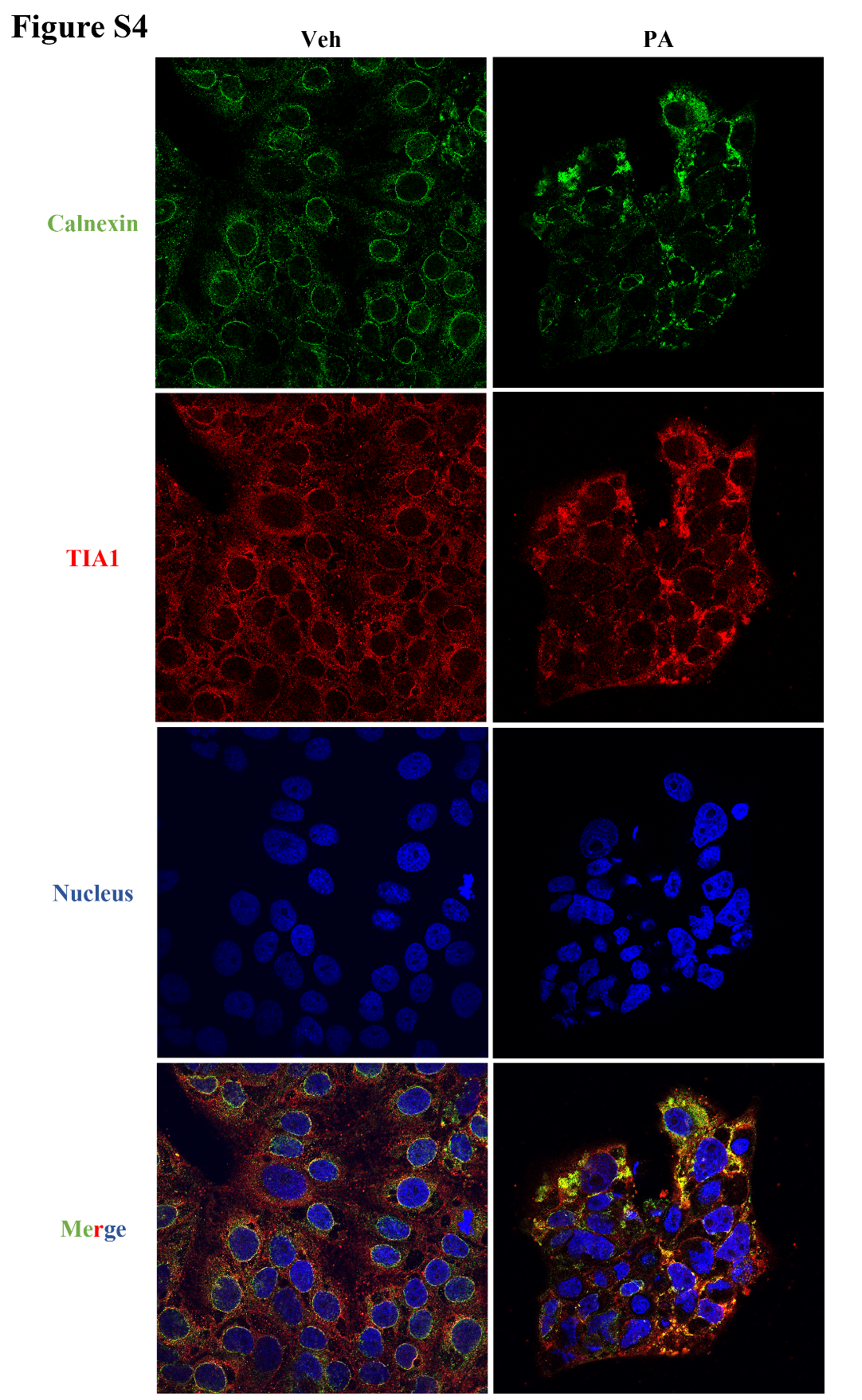


**Supplementary Fig S4: Calnexin and TIA1 are colocalized in PA treated JEG-3 cells.** JEG-3 cells were treated with either vehicle (Veh) or 400 μM PA for 24 hr and analyzed for calnexin (green) and TIA1 (red) using immunofluorescence. We observed increased co-localization of calnexin and TIA1 as assessed by increased appearance of orange/yellow points in PA treated cells compared to Veh treated cells. Cells were counterstained with DAPI (Nucleus).
